# Automated adaptive inference of phenomenological dynamical models

**DOI:** 10.1038/ncomms9133

**Published:** 2015-08-21

**Authors:** Bryan C. Daniels, Ilya Nemenman

**Affiliations:** 1 Center for Complexity and Collective Computation, Wisconsin Institute for Discovery, University of Wisconsin, Madison, Wisconsin 53715, USA; 2 Department of Physics, Emory University, Atlanta, Georgia 30322, USA; 3 Department of Biology, Emory University, Atlanta, Georgia 30322, USA

## Abstract

Dynamics of complex systems is often driven by large and intricate networks of microscopic interactions, whose sheer size obfuscates understanding. With limited experimental data, many parameters of such dynamics are unknown, and thus detailed, mechanistic models risk overfitting and making faulty predictions. At the other extreme, simple *ad hoc* models often miss defining features of the underlying systems. Here we develop an approach that instead constructs phenomenological, coarse-grained models of network dynamics that automatically adapt their complexity to the available data. Such adaptive models produce accurate predictions even when microscopic details are unknown. The approach is computationally tractable, even for a relatively large number of dynamical variables. Using simulated data, it correctly infers the phase space structure for planetary motion, avoids overfitting in a biological signalling system and produces accurate predictions for yeast glycolysis with tens of data points and over half of the interacting species unobserved.

## Abstract (web summary)

Mechanistic modelling of dynamical phenomena with many degrees of freedom runs the risk of overfitting and making faulty predictions, whereas *ad hoc* models may miss defining features. Here the authors develop an approach to construct dynamical models that adapt their complexity to the amount of available data.

## Graphical abstract

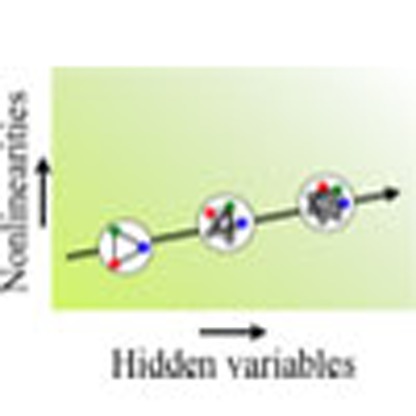

One can view the physics enterprise as reverse-engineering of Nature—using data to infer predictive mathematical models of physical systems, and then finding similarities among such models of distinct systems to identify physical laws. In the era of Big Data, these models are becoming Big Models, which are often as complicated as the data themselves, reflecting the humorous maxim that ‘the best material model of a cat is another, or preferably the same, cat'^1^. This is especially evident in modern biophysics and systems biology^2^, which are the primary focus of this article. Continued success of such approaches that systematize all known details in a combinatorially large mathematical model is uncertain. Indeed, generalizing and generating insight from complex models is difficult. Further, specification of myriads of microscopic mechanistic parameters in such models demands vast data sets and computational resources, and is hard even for very large data sets due to widely varying sensitivities of predictions to the parameters^3^. Finally, the very structures of these models are often unknown because they depend on many yet-unobserved players on the microscopic level. Identification of these structural characteristics is labour intensive and does not scale up easily. Thus, it is unlikely that mathematical models based solely on a detailed microscopic representation will be able to account accurately for the observed dynamics of many complex systems. More importantly, even if they could, the resulting models would be too unwieldy to bring about understanding of the modelled systems. Model reduction may alleviate some of these problems, but it still suffers from the difficulty of needing an exact, detailed model as an intermediate step^4^,^5^,^6^,^7^.

Because of these difficulties, the need to predict responses of complex systems to dynamical perturbations has led to a resurgence of research into automated inference of dynamical systems from time series data, which had been attempted since the early days of the field of nonlinear dynamics^8^,^9^. Approaches have been developed using linear dynamic models^10^, Bayesian Networks (Supplementary Note 8), recurrent neural networks^11^, evolved regulatory networks^12^ and symbolic regression^13^,^14^. The latter two produce models that are more mechanistically accurate and interpretable. However, because of the focus on microscopic accuracy, these approaches require searching through an extremely large space of all possible microscopic dynamics. In general, this leads to very long search times^12^,^14^, especially if some underlying variables are unobserved, and dynamics are coupled and cannot be inferred one variable at a time.

To move forward, we note that microscopic and macroscopic complexity are not necessarily related^15^,^16^. Thus, complex living systems may realize rather simple dynamics, at least in typical experimental setups. For example, activation of a combinatorially complex receptor can be specified with only a handful of effective parameters, including the dynamic range, cooperativity and time delay^17^,^18^,^19^, and the purpose of microscopic structural complexity can be in making the simple macroscopic functional output robust in the face of perturbations^18^,^20^. Similarly, in engineering^21^, effective models are often sufficient for forward (but not reverse) engineering of complex systems, as illustrated by the ubiquity of the purely phenomenological Kalman filter. These considerations suggest that macroscopic prediction does not necessarily require microscopic accuracy even in systems biology^22^, and that a complementary approach is needed, one in which we seek phenomenological, coarse-grained models of cellular processes that are simple and inferable, and nonetheless predictive and useful in limited domains^23^,^24^.

Here we propose an adaptive approach for inference of dynamics from time series data that does not attempt to find the single best microscopically ‘correct' model, but rather a phenomenological, effective model that is ‘as simple as possible, but not simpler' than needed to account for the experimental data. De-emphasizing microscopic accuracy means that we do not have to search through all possible microscopic dynamics, and we can focus on a much smaller hierarchy of models. By choosing a hierarchy that is nested and complete, we gain theoretical guarantees of statistical consistency, meaning the approach is able to adaptively fit any smooth dynamics with enough data, yet is able to avoid problems with overfitting that can happen without restrictions on the search space^25^. While similar complexity control methods are well established in statistical inference^26^ and in choosing a systems biology model for data from a finite set of models^27^,^28^,^29^, we believe that they have not been used yet in the context of inferring complex, nonlinear dynamics from an infinite, complete set of all possible dynamics. Importantly, this adaptive approach requires testing a number of models that scales only polynomially with the number of dynamical variables. Further, it uses computational resources that asymptotically scale linearly with the number of observations. This allows us to construct models with much smaller computational effort and fewer experimental measurements, even when many dynamical variables are unobserved. While our main goal is effective dynamical modelling in systems biology, our approach works for general physical dynamical systems. In fact, we call it *Sir Isaac* due to its success in discovering the law of universal gravity from simulated data.

## Results

### Model classes for dynamical inference

We seek a phenomenological model of dynamics in the form:


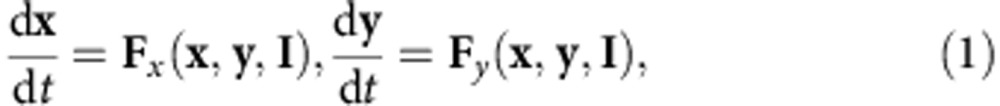


where **x** are observed variables, **y** are hidden variables and **I** are inputs or other parameters to the dynamics. We neglect intrinsic stochasticity in the dynamics (either deterministic chaotic or random thermal) and focus on systems for which repeated observations with nearly the same initial conditions produce nearly the same time series, save for measurement noise. The goal is then to find a phenomenological model of the force fields **F**_*x*_, **F**_*y*_ (ref. 8). The same dynamics may produce different classes of trajectories **x**(*t*) dependent on initial conditions (for example, ellipses and hyperbolas in gravitational motion). Dynamical inference rather than more familiar statistical modelling of trajectories is needed to represent these multiple functional forms within a single dynamical system.

Since our primary focus is on complex cellular processes, we construct two classes of nested and complete model hierarchies, both well matched to properties of biochemistry that underlies cellular network dynamics. We build the first with S-systems^30^ and the second with continuous time sigmoidal networks^31^. The S-systems use production and degradation terms for each dynamical variable formed by products of powers of all involved variables (chemical species concentrations); this is a natural generalization of biochemical mass–action laws. Specifically, an S-system consists of *J* dynamical variables *x*_*i*_ and *K* inputs *I*_*k*_=*x*_*J*+*k*_, with each dynamical variable governed by an ordinary differential equation (ref. 30)


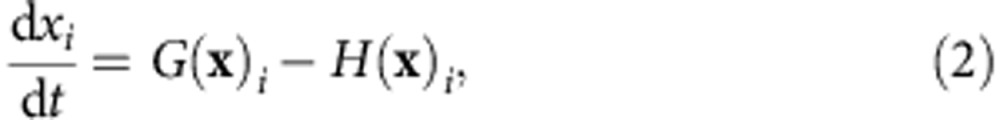


where production 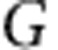 and degradation 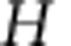 terms have the form:


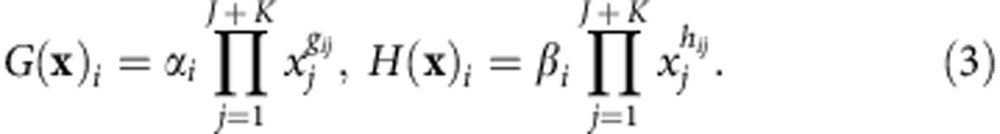


Second, the sigmoidal class represents interactions using linear combinations of saturating functions of species concentrations, similar to saturation in biochemical reaction rates:


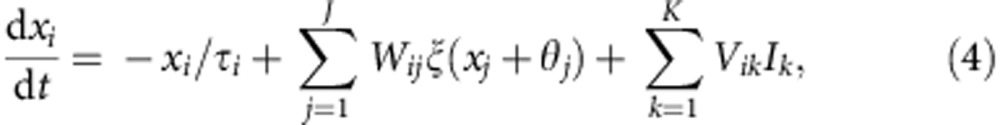


where the sigmoidal function *ξ*(*y*)=1/(1+*e*^*y*^). Importantly, both classes are complete and are able to represent any smooth, nonlinear dynamics with a sufficient number of (hidden) dynamical variables^30^,^32^,^33^. They can also each efficiently represent the types of sharp nonlinearities typically found in biophysical systems (Supplementary Note 1).

To perform adaptive fitting within a model class, a specific ordered hierarchy of models is chosen *a priori* that simultaneously varies both the degree of nonlinearity (the number of factors in equation 3 or terms in equation 4) and the number of hidden variables (additional *x*_*i*_; Supplementary Fig. 1 and Supplementary Note 1). Within this restricted model space, Bayesian inference is then used to select a single best model (see Methods section).

### The law of gravity

Before applying the approach to complex dynamics where the true model may not be expressible simply within the chosen search hierarchy, we test it on a simpler system with a known exact solution. We choose the iconic law of gravity, inferred by Newton based on empirical observations of trajectories of planets, the Moon and, apocryphally, a falling apple. Crucially, the inverse-squared distance law of Newtonian gravity can be represented exactly within the S-systems power-law hierarchy for elliptical and hyperbolic trajectories, which do not go to zero radius in finite time. It requires a hidden variable, the velocity, to completely specify the dynamics of the distance of an object from the sun (Supplementary Note 2).

**Figure 1:**
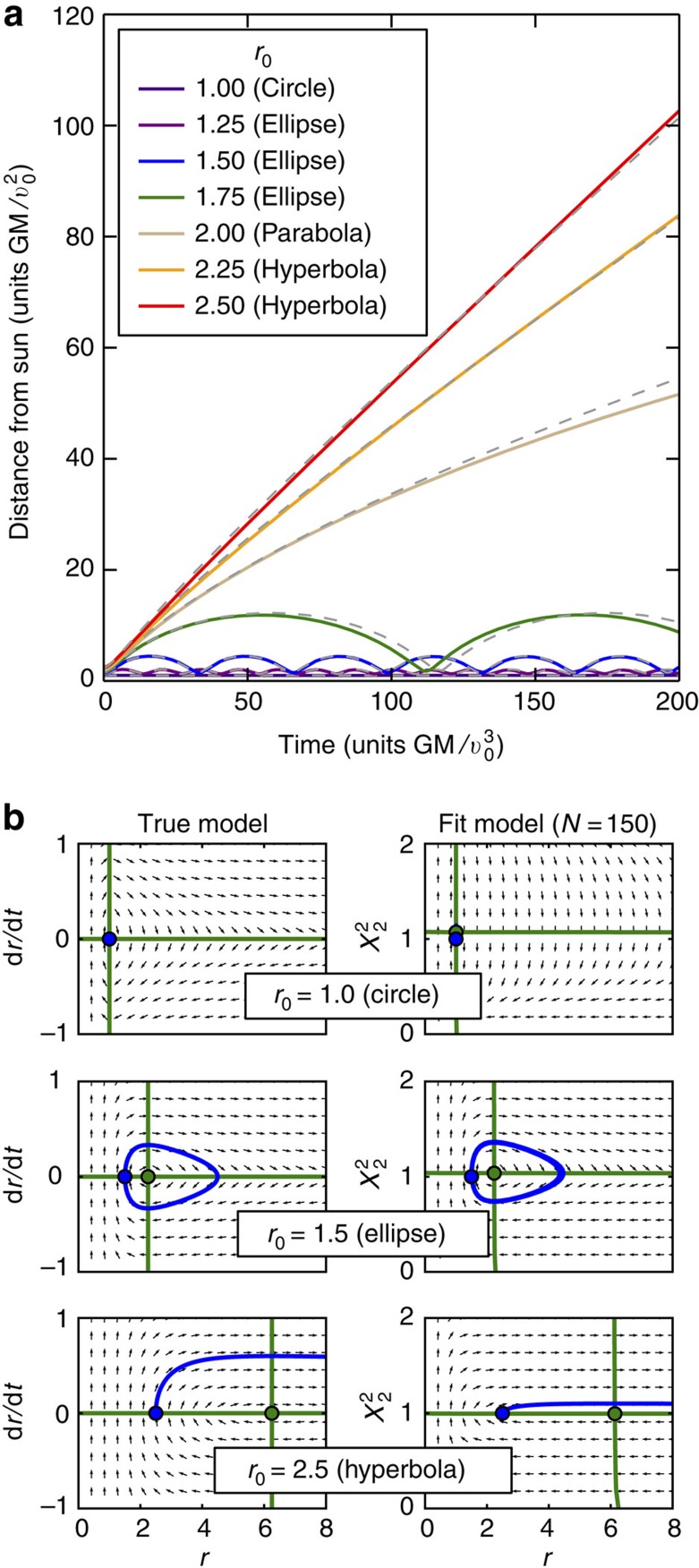
Dynamical inference of the law of gravity. A particle is released with velocity *v*_0_ perpendicular to the line connecting it to the sun, with varying initial distance *r*_0_ from the sun. (**a**) With only *N*=150 examples (each consisting of just a single noisy observation of *r* at a random time *t* after the release; Supplementary Note 2), we infer a single dynamical model in the S-systems class that reproduces the data. With no supervision, adaptive dynamical inference produces bifurcations that lead to qualitatively different behaviour: in this case, a single model produces both oscillations (elliptical orbits) and monotonic growth (hyperbolic trajectories). Inferred trajectories are shown with solid coloured lines, and the corresponding true trajectories are shown with dashed lines. (**b**) Similar to the true model (left), the inferred model (right) contains a single hidden variable *X*_2_ and works using a similar phase space structure. Specifically, the location of nullclines (green lines) and a single fixed point (green circle) as a function of *r*_0_ are recovered well by the fit. Note that the hidden variable is defined up to a power (Supplementary Note 2), and we choose to plot 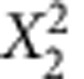 here.

Figure 1 displays the result of adaptive inference using the S-systems class. Given data about the distance of an object from the sun over time, the algorithm discovers a model that reproduces the underlying dynamics, including the necessary hidden variable and the bifurcation points. Since the trajectories include hyperbolas and ellipses, this example displays the advantage of inferring a single set of dynamical equations of motion, rather than statistical fits to trajectories themselves, which would be different in the two cases. This adaptive dynamical inference is comparable to other recent methods^13^, and it successfully treats a hidden dynamical variable. Supplementary Fig. 4 additionally shows inference of the law of gravity using the sigmoidal model class. While accurate, the fits are worse than those using S-systems, illustrating the importance of understanding basic features of the studied system when conducting automated model inference.

Empowered by the success of the adaptive inference approach in this case, we chose to name it *Sir Isaac*. The software implementation can be found under this name on GitHub.

### Multisite phosphorylation model

When inferring models for more general systems, we do not expect the true dynamics to be perfectly representable by any specific model class: even the simplest biological phenomena may involve combinatorially many interacting components. Yet for simple macroscopic behaviour, we expect to be able to use a simple approximate model that can produce useful predictions. To demonstrate this, we envision a single immune receptor with *n* modification sites, which can exist in 2^*n*^ microscopic states^34^, yet has simple macroscopic behaviour for many underlying parameter combinations. Here we test a model receptor that can be phosphorylated at each of *n*=5 sites arranged in a linear chain. The rates of phosphorylation and dephosphorylation at each site are affected by the phosphorylation states of its nearest neighbouring sites. With Michaelis–Menten kinetics and independence of kinetic rates for different states, this produces a complicated model with 32 coupled Ordinary Differential Equations (ODEs) specified by 52 parameters, which we assume are unknown to the experimenter.

We imagine an experimental setup in which we can control one of these parameters, for example, by changing concentrations of various kinases. We are interested in effects of such changes on the time evolution of the total phosphorylation of all five sites. Here we arbitrarily treat as input *I* the maximum rate of cooperative phosphorylation of site 2 due to site 3 being occupied, *V*. This is inspired, for example, by being able to measure or control concentrations of the SRC-family kinases (input), which mediate immune signalling conditional on the previous steps in the receptor activation sequence being completed^17^. We then ‘measure' the resulting time course of total phosphorylation starting from the unphosphorylated state. Experimental measurements are corrupted with noise at the scale of 10% of their values (see Supplementary Note 3 for details).

A straightforward approach to modelling this system is to fit the 52 parameters of the known model to the data. A second approach is to rely on intuition to manually develop a functional parameterization that captures the most salient features of the time course data. In this case, we can write a simple 5-parameter model (Supplementary Note 3) that captures exponential saturation in time with an asymptotic value that depends sigmoidally on the input *V*. A third approach, advocated here, is to use automated model selection to create a model with complexity that matches the amount and precision of the available data.

**Figure 2:**
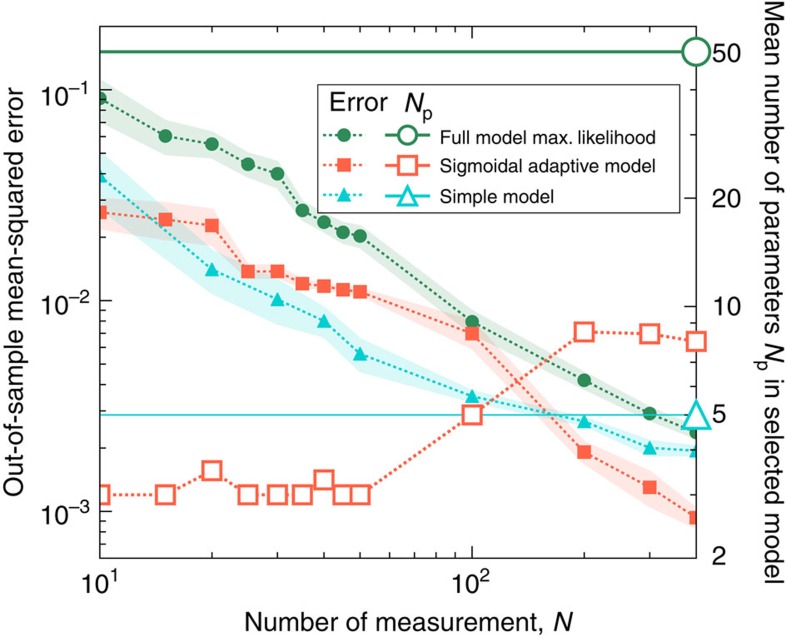
Multisite phosphorylation model selection as a function of the number of measurements *N*. The sizes of errors made by three models (filled symbols; left axis) decrease as the amount of data increases. Adaptive sigmoidal models (orange squares) outperform a maximum (max.) likelihood fit to the full 52-parameter model (green circles) in this range of *N* (although we expect that it will eventually outperform all other models as *N*→∞). A simple 5-parameter model (blue triangles) that is custom-made to match salient features of the true behaviour is the best performer for a moderate amount of data, but is outperformed by adaptive models when given more data. The mean over 10 sets of input data are shown, with shaded regions indicating the s.d. of the mean. The full and simple models each use a fixed number of parameters (open symbols; right axis), while the sigmoidal model adapts to use more parameters when given more data.

**Figure 3:**
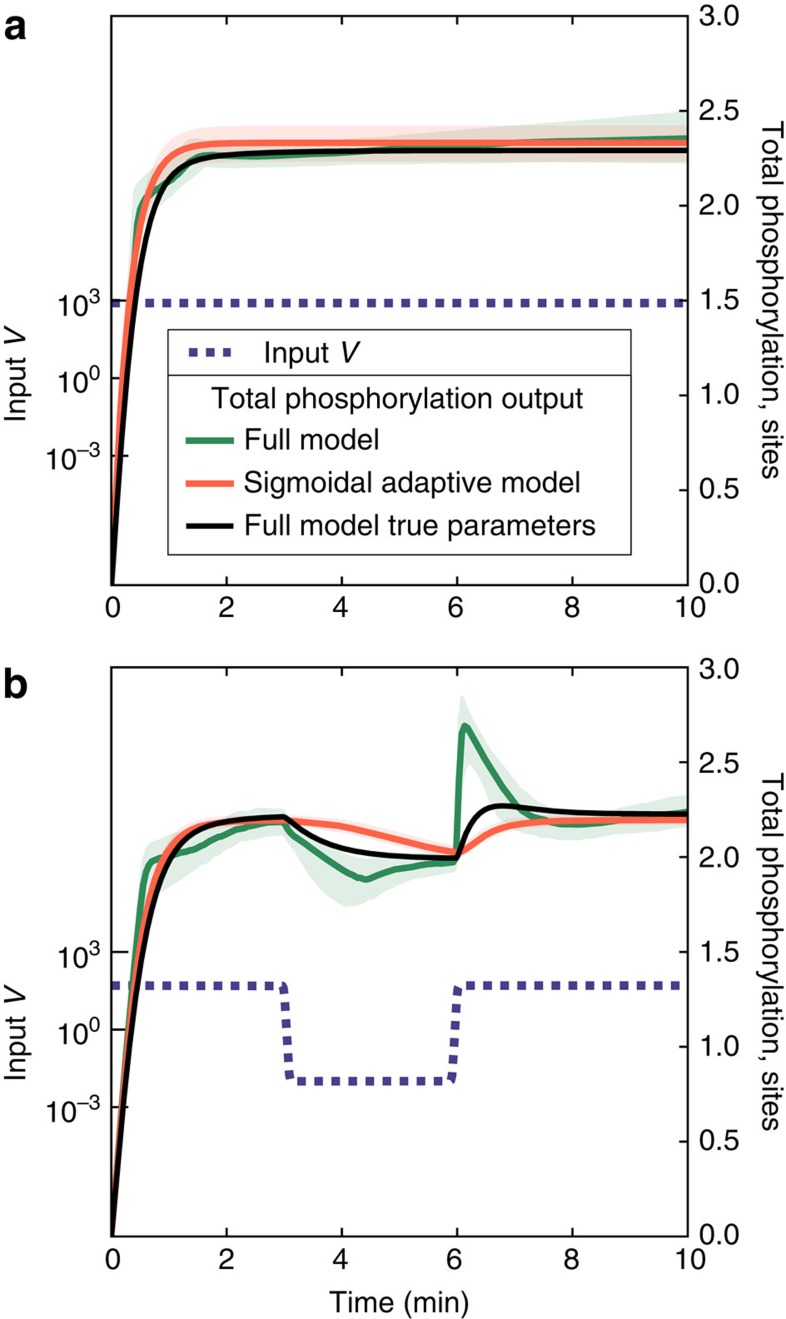
Time series responses to out-of-sample inputs in inferred models of multisite phosphorylation. Plotted is the predicted response (right axis) to (**a**) constant and (**b**) time-varying input (left axis, blue lines). Fit to *N*=300 constant input data points, the full-known model (green) produces erratic behaviour typical of overfitting (especially evident in **b**), while the adaptive sigmoidal model (orange) produces more stable out-of-sample predictions with median behaviour that is closer to the true dynamics. Plotted is the median behaviour over 100 samples from each model's parameter posterior (Supplementary Note 3), with shaded regions indicating 90% confidence intervals, which are in some cases smaller than the width of the line.

In Fig. 2, we compare these three approaches as the amount of available data is varied, and Fig. 3a shows samples of fits done by different procedures. With limited and noisy data, fitting the parameters of the full known model risks overfitting, and in the regime we test, it is the worst performer on out-of-sample predictions. The simple model performs best when fitting to <100 data points, but for larger amounts of data it saturates in performance, as it cannot fit more subtle effects in the data. In contrast, an adaptive model remains simple with limited data and then grows to accommodate more subtle behaviours once enough data are available, eventually outperforming the simple model. Even when given up to 400 data points, the adaptive model remains relatively simple, avoiding using as many degrees of freedom as the full model (Supplementary Fig. 5). Crucially, this performance stays robust when various assumptions of the adaptive inference approach are violated (such as the model of the measurement noise, cf. Supplementary Fig. 2A,B). And it barely depends on details of the approach such as the ordering with which parameters are added into the model (cf. Supplementary Fig. 2C).

The multisite phosphorylation example also demonstrates that dynamical phenomenological models found by *Sir Isaac* are more than fits to the existing data, but rather they uncover the true nature of the system in a precise sense: they can be used to predict responses to some classes of inputs that are qualitatively different from those used in the inference. For example, as seen in Fig. 3b, an adaptive sigmoidal model inferred using temporally constant signals produces a reasonable extrapolated prediction for response to a time-varying signal. At the same time, overfitting is evident when using the full, detailed model, even when one averages responses over the posterior distribution of the inferred model parameters.

### Yeast glycolysis model

**Figure 4:**
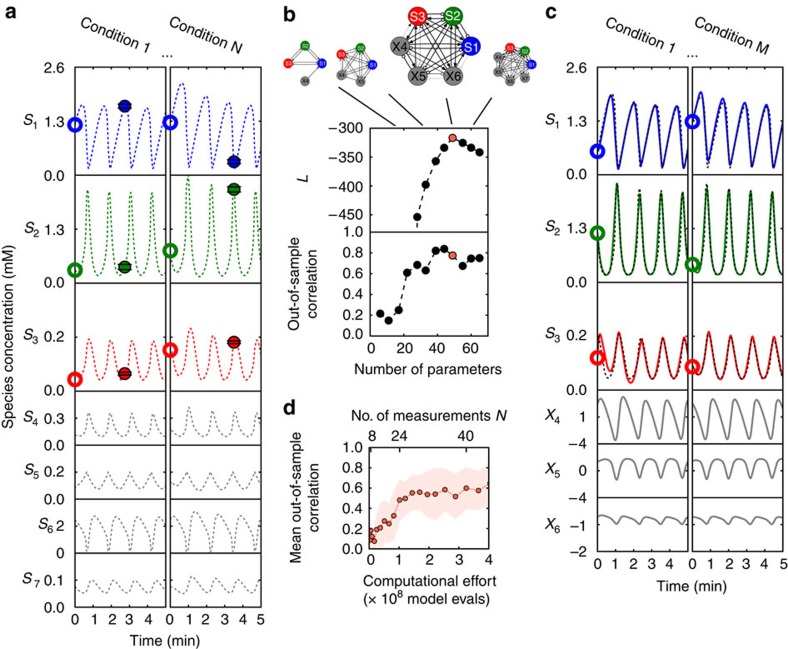
The model selection process using time course measurements of three metabolites in yeast glycolysis. (**a**) For each set of given initial conditions (open circles), a noisy measurement of the three observable concentrations (filled circles) is made at a single random time. Hidden variables (in grey) are not measured. In this example, we fit to *N*=40 in-sample conditions. (**b**) Models from an ordered class, with the illustrated connectivity, are fit and tested sequentially until 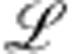, an approximation of the relative log-likelihood, decreases sufficiently from a maximum. (**c**) The selected model (large connectivity diagram) is used to make predictions about out-of-sample conditions. Here, we compare the output of the selected model (solid lines) with that of the model that created the synthetic data (dashed lines). (**d**) Performance versus computational and experimental effort. The mean out-of-sample correlation for three measured biochemical species from the range of initial conditions twice that used in training rises to over 0.6 using <5 × 10^8^ model evaluations and 40 in-sample measurements. In (ref. 14), inferring an exact match to the original seven-dimensional model used roughly 500 times as many measurements of all seven species (with none hidden). The approach also used 200 times as many model evaluations (Supplementary Note 4). Nonetheless, the accuracy of both approaches is comparable, and *Sir Isaac* additionally retains information about the phase of the oscillations. This illustrates that the problem of adaptively finding an approximation to the dynamics is, in fact, much simpler than the problem of inferring the detailed equations describing the dynamics.

A more complicated test of the method is to reproduce nonlinear oscillatory dynamics, such as that describing yeast glycolysis, for which there has been recent interest in automated inference^14^,^24^. A recent model for the system^35^,^36^, informed by detailed knowledge of metabolic pathways, consists of coupled ODEs for seven species whose concentrations oscillate with a period near 1 min. The system dynamic is simpler than its structure in the sense that some complexity is used to stabilize oscillations to perturbations. On the other hand, the oscillations are not smooth (Fig. 4) and hence are hard to fit with simple methods. These aspects make this model an ideal test case for *Sir Isaac*.

If we were given abundant time series data from all seven species and were confident that there were no other important hidden species, we may be in a position to infer a ‘true' model detailing interactions among them. If we are instead in the common situation of having limited data on a limited number of species, we may more modestly attempt to make predictions about the inputs and outputs that we have measured. This is conceptually harder since an unknown number of hidden variables may need to be introduced to account for the dynamics of the observed species. We demonstrate our approach by constructing adaptive models using data for only three of the seven coupled chemical species, as their initial conditions are varied.

Depicted in Fig. 4 is the model selection procedure for this case. After selecting an adaptive model fit to noisy data from *N* single time points, each starting from initial conditions sampled from specified ranges, we test the inferred model's ability to predict the time course resulting from out-of-sample initial conditions, including those lying far away from the limit cycle. With data from only *N*=40 measurements, the selected model is able to predict behaviour with mean correlation of over 0.6 for initial conditions chosen from ranges twice as large as those used as training data (shown in Fig. 4) and 0.9 for out-of-sample ranges equal to in-sample ranges (shown in Supplementary Fig. 9). At this point, the model saturates at ∼65 nominal and 35 effective parameters (Supplementary Fig. 11). This is larger than in the true model and does not necessarily reflect its topology. However, since discovering the functional form of the true model (including hidden nodes) would require a search through a much larger space of models, complexity here should not be measured just by the number of parameters. This is illustrated, in part, by the admirable predictive performance of the phenomenological model for a relatively small *N*.

We can compare this to the performance of a hand-constructed ‘simple' 9-parameter harmonic oscillator model (an analogue of the simple model in the multisite phosphorylation case). The simple model, for which the numbers of nominal and effective parameters are equal (Supplementary Fig. 11), does not have the exploratory power to resolve the sharp peaks and obtain good predictions (Supplementary Note 4 and Supplementary Fig. 9). In another comparison, the true model that generated the data has 16 parameters, which is fewer than the result of *Sir Isaac*. However, the functional form of the dynamics for this exact model should also be counted as inferred parameters, making such comparisons harder. In fact, because of this, previous work that inferred the exact equations of the original seven-dimensional model (including also an unexpected conservation law)^14^ had to use roughly 500 times as many measurements of all 7 variables and 200 times as many model evaluations. While *Sir Isaac* is somewhat aided by an appropriate choice of sigmoidal basis functions, and has not been designed to look for conservation laws, this example illustrates how focusing on a simpler problem, namely, finding an approximate, phenomenological model of the process, can decrease data requirements by orders of magnitude. This example also demonstrates that adaptive modelling can hint at the complexity of the hidden dynamics beyond those measured: the best performing sigmoidal model requires three hidden variables, for a total of six chemical species, which is exactly what one would expect for a seven-dimensional system with a (hidden) conservation law^14^. Crucially, the computational complexity of *Sir Isaac* still scales linearly with the number of observations, even when a large fraction of variables remains hidden (Supplementary Note 7 and Supplementary Fig. 10). We anticipate that using advanced approaches to identify and conduct the most informative experiments and efficiently search the model hierarchy using genetic algorithms, as in (ref. 14), may improve performance further.

## Discussion

The three examples demonstrate the power of the adaptive, phenomenological dynamical modelling approach. *Sir Isaac* models are inferred without an exponentially complex search over model space, which would be impossible for systems with many variables. These models are as simple or complex as warranted by data and are guaranteed not to overfit even for small data sets. Thus, they require orders of magnitude less data and computational resources to achieve the same predictive accuracy as methods that infer a pre-defined, large number of mechanistic parameters in the true model description.

These advantages require that the inferred models are phenomenological and are designed for efficiently predicting system dynamics at a given scale, determined by the available data. While Fig. 1 shows that *Sir Isaac* will infer the true model if it is within the searched model hierarchy and enough data are available, more generally the inferred dynamics may be quite distinct from the true microscopic mechanisms, as shown by a different number of chemical species in the true and the inferred dynamics in Fig. 4. What is then the utility of the approach if it says little about underlying mechanisms?

First, there is the obvious advantage of being able to predict responses of systems to yet-unseen experimental conditions, including those qualitatively different from the ones used for inference. This is trivially useful in the context of engineering and control, where predictive, usable models are often necessarily far removed from microscopic precision^21^. Second, some general mechanisms, such as the necessity of feedback loops or hidden variables, are easily uncovered even in phenomenological models. However, more importantly, we draw the following analogy. When in the seventeenth century Robert Hooke studied the force-extension relations for springs, a linear model for a specific spring did not tell much about the force generation. However, the observation that all springs exhibit such linear relations for small extensions allowed him to combine the models into a law—Hooke's law—the first of many phenomenological physical laws that followed. It instantly became clear that experimentally measuring just one parameter, the Hookean stiffness, provided an exceptionally precise description of the spring's behaviour. And yet the mechanistic understanding of how this Hooke's constant is related to atomic interactions within materials is only now starting to emerge. Similarly, by studying related phenomena across complex living systems (for example, chemotactic behaviour in *Escherichia coli*^37^ and *Caenorhabditis elegans*^38^, or behavioural bet hedging, which can be done by a single-cell^39^ or a behaving rodent^40^), we hope to build enough models of specific systems, so that general physical laws describing how nature implements them become apparent.

If successful, our search for phenomenological, emergent dynamics should allay some of the most important scepticism regarding the utility of automated dynamical systems inference in science^41^, namely, that such methods typically start with known variables of interest and known underlying physical laws, and hence cannot do transformative science and find new laws of nature. Indeed, we demonstrated that, for truly successful predictions, the model class used for automated phenomenological inference must match basic properties of the studied dynamics (contrast, for example, Fig. 1 with Supplementary Fig. 4, and see Supplementary Fig. 6). Thus, fundamental properties of the underlying mechanisms, such as the power-law structure of the law of gravity or the saturation of biochemical kinetic rates, can be inferred from data even if unknown *a priori*. Finally, we can contrast our approach with a standard procedure for producing coarse-grained descriptions of physical systems: starting from mechanistically accurate dynamics, and then mapping them onto one of a small set of universality classes^22^,^42^. This procedure is possible due to symmetries of physical interactions that are not typically present in living systems. Without such symmetries, the power of universality is diminished, and different microscopic models may result in similarly different macroscopic ones. Then specifying the microscopic model to coarse grain it later becomes an example of solving a harder problem to solve a simpler one^43^. Thus, for living systems, the direct inference of phenomenological dynamics, such as done by *Sir Isaac*, may be the optimal way to proceed.

## Methods

### Data and software availability

All simulated data used in this paper, as well as data analysis and plotting scripts, are available at http://dx.doi.org/10.6084/m9.figshare.1491421. Additional instructions on how to work with the data can be found in the archive. The Python-based software implementation of Sir Isaac is available from https://github.com/EmoryUniversityTheoreticalBiophysics/SirIsaac.

### Classes of phenomenological models used by *Sir Isaac*

To create a model in the form of Eq. (1), we would like to gradually increase the complexity of *F* until we find the best tradeoff between good fit and sufficient robustness, essentially extending traditional Bayesian model selection techniques to the realm of an infinite set of possible dynamical models. Ideally, this process should progress similarly to a Taylor series approximation to a function, adding terms one at a time in a hierarchy from simple to more complex, until a desired performance is obtained. To guarantee that this is possible, the hierarchy of models must be nested (or ordered) and complete in the sense that any possible dynamics can be represented within the hierarchy^25^ (Supplementary Note 1). Any model hierarchy that fits these criteria may be used, yet specification of the hierarchy is nontrivial in that it requires choosing an ordering of models that gradually adds both nonlinearities and unobserved variables. Further, different model hierarchies may naturally perform differently on the same data, depending on whether the studied dynamics can be represented succinctly within a hierarchy. Our results suggest that the choice of model class, specifying the functional forms used to model the dynamics, is more important to performance than the subsequent choice of the order of adding parameters within that class (Supplementary Fig. 2C).

Our first model class is the S-system power-law class, defined in equations 2 and 3. In a process called ‘recasting', any set of differential equations written in terms of elementary functions can be rewritten in the power-law form by defining new dynamical variables in the correct way^30^. Since any sufficiently smooth function can be represented in terms of a series of elementary functions (for example, Taylor series), a power-law network of sufficient size can describe any such deterministic dynamical system. Note that, since exponents are not constrained to be positive or integer-valued, dynamics in this class are generally ill-defined when variables are not positive. We find that the S-systems model class works well for planetary motion, which has an exact representation in the class (Supplementary Note 2). For our biological test examples, the S-systems class is outperformed by the sigmoidal class (see below). This may be indicating that behaviour common in the S-systems class is not common in typical biological systems (for example, real production and degradation terms cannot grow without bounds). It may also stem from the positivity constraint: since the condition that variables remain positive is not easily determined from parameter values, we are forced in our model selection process to simply discard any tested parameters that lead to zero or negative values.

The second model hierarchy that we construct is the sigmoidal network class. In this class, we use the fact that the interactions among biological components often take the form of a sigmoidal function to define the system of ODEs in equation 4. This class of models has also been shown to approximate any smooth dynamics arbitrarily well with a sufficient number of dynamical variables^31^,^32^,^33^,^44^. Note that natural variations of this class to be explored in future work include rescaling of the arguments of the sigmoids *ξ* or switching the order of operations to apply the sigmoidal function to a linear combination of state variables to more closely match traditional neural network models^45^.

It is possible that both the S-system and sigmoidal classes can be unified into power-law dynamical systems with algebraic power-law constraints among the dynamical variables^30^, but this will not be explored in this report. Other than these two model classes and their modifications described above, the authors are not aware of other biologically relevant dynamical representations that are currently known to be complete. Yet others certainly exist and could be developed into alternate model hierarchies in future work.

### Description of model selection procedure

For each model in the hierarchy, its parameters are fit to the data using a two-step process akin to simulated annealing (Supplementary Note 6), with best-fit parameters from the next simplest model in the hierarchy used as a starting point to speed convergence. The resulting fit model is evaluated by calculating an estimate of the Bayesian log-likelihood 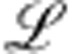. This estimate makes use of a generalized version of the Bayesian Information Criterion^46^, which is described in detail in Supplementary Note 5. We believe that this is the first time the Bayesian Information Criterion has been adopted for use with automated nonlinear dynamical systems inference over an infinite set of models. As models increase in complexity, 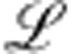 first grows as the quality of fit increases, but eventually begins to decrease, signifying overfitting. Since, statistical fluctuations aside, there is just one peak in 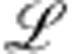 (ref. 25), one can be certain that the global maximum has been observed once it has decreased sufficiently. The search through the hierarchy is then stopped, and the model with maximum 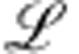 is ‘selected' (Fig. 4b).

## Additional information

**How to cite this article:** Daniels, B. C. & Nemenman, I. Automated adaptive inference of phenomenological dynamical models. *Nat. Commun.* 6:8133 doi: 10.1038/ncomms9133 (2015).

## Supplementary Material

Supplementary Information Supplementary Figures 1-11, Supplementary Tables 1-5, Supplementary Notes 1-8 and Supplementary References

## References

[b1] Rosenblueth A. & Wiener N. The role of models in science. Philos. Sci. 12, 316–321 (1945).

[b2] Hlavacek W. How to deal with large models? Mol. Syst. Biol. 5, 240 (2009). 19156132 10.1038/msb.2008.80 PMC2644176

[b3] Gutenkunst R. *et al.* Universally sloppy parameter sensitivities in systems biology models. PLoS Comput. Biol. 3, 1871–1878 (2007). 17922568 10.1371/journal.pcbi.0030189 PMC2000971

[b4] Feret J., Danos V., Krivine J., Harmer R. & Fontana W. Internal coarse-graining of molecular systems. Proc. Natl Acad. Sci. USA 106, 6453–6458 (2009). 19346467 10.1073/pnas.0809908106 PMC2672529

[b5] Borisov N., Chistopolsky A., Faeder J. & Kholodenko B. Domain-oriented reduction of rule-based network models. IET Syst. Biol. 2, 342–351 (2008). 19045829 10.1049/iet-syb:20070081 PMC2628550

[b6] Danos V., Feret J., Fontana W., Harmer R. & Krivine J. in Logic in Computer Science 362–381IEEE Computer Society (2010).

[b7] Dokoumetzidis A. & Aarons L. Proper lumping in systems biology models. IET Syst. Biol. 3, 40–51 (2009). 19154083 10.1049/iet-syb:20070055

[b8] Crutchfield J. & McNamara B. Equations of motion from a data series. Complex Syst. 1, 417–452 (1987).

[b9] Packard N., Crutchfield J., Farmer J. & Shaw R. Geometry from a time series. Phys. Rev. Lett. 45, 712–716 (1980).

[b10] Friston K., Harrison L. & Penny W. Dynamic causal modelling. NeuroImage 19, 1273–1302 (2003). 12948688 10.1016/s1053-8119(03)00202-7

[b11] Sussillo D. & Abbott L. F. Generating coherent patterns of activity from chaotic neural networks. Neuron 63, 544–557 (2009). 19709635 10.1016/j.neuron.2009.07.018 PMC2756108

[b12] François P., Hakim V. & Siggia E. D. Deriving structure from evolution: metazoan segmentation. Mol. Syst. Biol. 3, 154 (2007). 18091725 10.1038/msb4100192 PMC2174625

[b13] Schmidt M. & Lipson H. Distilling free-form natural laws from experimental data. Science 324, 81–85 (2009). 19342586 10.1126/science.1165893

[b14] Schmidt M. *et al.* Automated refinement and inference of analytical models for metabolic networks. Phys. Biol. 8, 055011 (2011). 21832805 10.1088/1478-3975/8/5/055011 PMC4109817

[b15] Anderson P. W. More is different. Science 177, 393–396 (1972). 17796623 10.1126/science.177.4047.393

[b16] May R. Simple mathematical models with very complicated dynamics. Nature 261, 459–467 (1976). 934280 10.1038/261459a0

[b17] Goldstein B., Faeder J. & Hlavacek W. Mathematical and computational models of immune-receptor signalling. Nat. Rev. Immunol. 4, 445–456 (2004). 15173833 10.1038/nri1374

[b18] Bel G., Munsky B. & Nemenman I. The simplicity of completion time distributions for common complex biochemical processes. Phys. Biol. 7, 016003 (2010). 20026876 10.1088/1478-3975/7/1/016003

[b19] Cheong R., Rhee A., Wang, Nemenman I. & Levchenko A. Information transduction capacity of noisy biochemical signaling networks. Science 334, 354–358 (2011). 21921160 10.1126/science.1204553 PMC3895446

[b20] Lander A. Pattern, growth, and control. Cell 144, 955–969 (2011). 21414486 10.1016/j.cell.2011.03.009 PMC3128888

[b21] LeDuc P. R., Messner W. C. & Wikswo J. P. How do control-based approaches enter into biology? Annu. Rev. Biomed. Eng. 13, 369–396 (2011). 21599491 10.1146/annurev-bioeng-071910-124651

[b22] Machta B. B., Chachra R., Transtrum M. K. & Sethna J. P. Parameter space compression underlies emergent theories and predictive models. Science 342, 604–607 (2013). 24179222 10.1126/science.1238723

[b23] Wiggins C. & Nemenman I. Process pathway inference via time series analysis. Exp. Mech. 43, 361–370 (2003).

[b24] Daniels B. C. & Nemenman I. Efficient inference of parsimonious phenomenological models of cellular dynamics using S-systems and alternating regression. PLoS ONE 10, e0119821 (2015). 25806510 10.1371/journal.pone.0119821 PMC4373916

[b25] Nemenman I. Fluctuation-dissipation theorem and models of learning. Neural. Comput. 17, 2006–2033 (2005). 15992488 10.1162/0899766054322982

[b26] MacKay D. Information theory, inference, and learning algorithms. (Cambridge Univ. Press (2003).

[b27] Vyshemirsky V. & Girolami M. Bayesian ranking of biochemical system models. Bioinformatics 24, 833–839 (2008). 18057018 10.1093/bioinformatics/btm607

[b28] Toni T., Welch D., Strelkowa N., Ipsen A. & Stumpf M. Approximate Bayesian computation scheme for parameter inference and model selection in dynamical systems. J. R Soc. Interface 6, 187–202 (2009). 19205079 10.1098/rsif.2008.0172 PMC2658655

[b29] Lillacci G. & Khammash M. Parameter estimation and model selection in computational biology. PLoS Comput. Biol. 6, e1000696 (2010). 20221262 10.1371/journal.pcbi.1000696 PMC2832681

[b30] Savageau M. A. & Voit E. O. Recasting nonlinear differential equations as S-Systems: a canonical nonlinear form. Math. Biosci. 87, 83–115 (1987).

[b31] Beer R. D. Parameter space structure of continuous-time recurrent neural networks. Neural Comput. 18, 3009–3051 (2006). 17052157 10.1162/neco.2006.18.12.3009

[b32] Funahashi K.-I. & Nakamura Y. Approximation of dynamical systems by continuous time recurrent neural networks. Neural Networks 6, 801–806 (1993).

[b33] Chow T. W. & Li X.-D. Modeling of continuous time dynamical systems with input by recurrent neural networks. IEEE Trans. Circ. Syst. I Fundam. Theory Appl. 47, 575–578 (2000).

[b34] Hlavacek W. S. *et al.* Rules for modeling signal-transduction systems. Sci. STKE 2006, re6 (2006). 16849649 10.1126/stke.3442006re6

[b35] Wolf J. & Heinrich R. Effect of cellular interaction on glycolytic oscillations in yeast: a theoretical investigation. Biochem. J. 334, 321–334 (2000). 10702114 PMC1220770

[b36] Ruoff P., Christensen M., Wolf J. & Heinrich R. Temperature dependency and temperature compensation in a model of yeast glycolytic oscillations. Biophys. Chem. 106, 179–192 (2003). 14556906 10.1016/s0301-4622(03)00191-1

[b37] Berg H. E. coli in Motion Springer (2004).

[b38] Ryu W. & Samuel A. Thermotaxis in Caenorhabditis elegans analyzed by measuring responses to defined thermal stimuli. J. Neurosci. 22, 5727–5733 (2002). 12097525 10.1523/JNEUROSCI.22-13-05727.2002 PMC6758190

[b39] Kussell E. & Leibler S. Phenotypic diversity, population growth, and information in fluctuating environments. Science 309, 2075–2078 (2005). 16123265 10.1126/science.1114383

[b40] Gallistel C., Mark T., King A. & Latham P. The rat approximates an ideal detector of changes in rates of reward: implications for the law of effect. J. Exp. Psychol. Anim. Behav. Process 27, 354–372 (2001). 11676086 10.1037//0097-7403.27.4.354

[b41] Anderson P. W. & Abrahams E. Machines fall short of revolutionary science. Science 324, 1515–1516 (2009). 19541975 10.1126/science.324_1515c

[b42] Wilson K. Renormalization group and critical phenomena. I. Renormalization group and the Kadanoff scaling picture. Phys. Rev. B 4, 3174–3183 (1971).

[b43] Vapnik V. The Nature of Statistical Learning Theory 2nd, edn Springer (2000).

[b44] Beer R. D. & Daniels B. Saturation probabilities of continuous-time sigmoidal networks. Preprint at http://arxiv.org/abs/1010.1714 (2010).

[b45] Rumelhart D., Hinton G. & Williams R. Learning representations by back-propagating errors. Nature 323, 533–536 (1986).

[b46] Schwarz G. Estimating the dimension of a model. Annal. Stat. 6, 461–464 (1978).

