# Supplementary material for: Automated adaptive inference of phenomenological dynamical models
Source: Nat Commun. 2015 Aug 21;6:8133. doi: 10.1038/ncomms9133 (PMC4560822; doi:10.1038/ncomms9133)
Supplement: Supplementary Information — Supplementary Figures 1-11, Supplementary Tables 1-5, Supplementary Notes 1-8 and Supplementary References [file ncomms9133-s1.pdf]

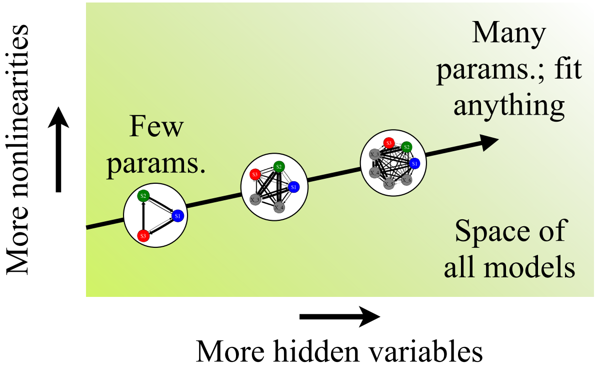

Supplementary Figure 1. Hierarchical model selection follows a single predefined path through model space.

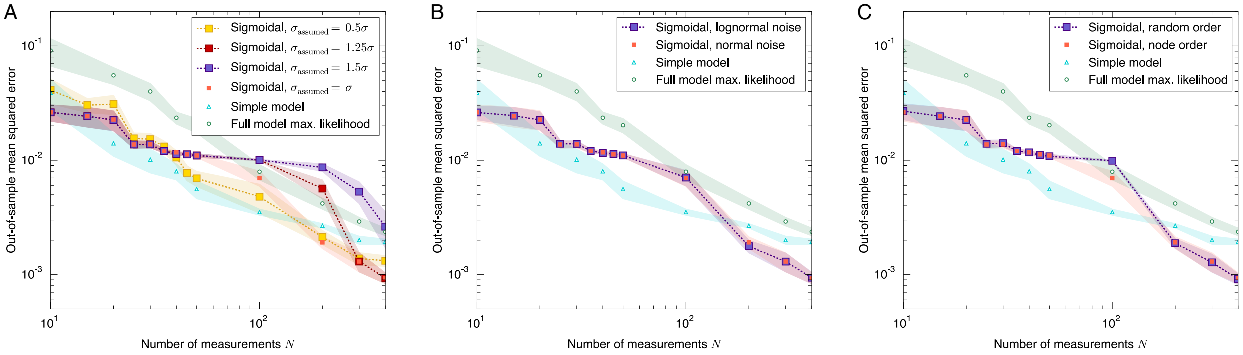

Supplementary Figure 2. Testing the robustness of adaptive inference in the multi-site phosphorylation example. In each case, the original performance curves from the main text's Figure 2 (smaller symbols) are compared to an altered version of the model selection process (larger symbols). (A) Comparing fitting to the same data but using an incorrect standard deviation $\sigma_{\rm assumed}$ when calculating the Bayesian log-likelihood. (B) Comparing fitting to data with log-normally distributed noise; the two lines overlap and are hard to distinguish on the plot. (C) Comparing to adding parameters in random order, averaged over 10 realizations. See text for details.

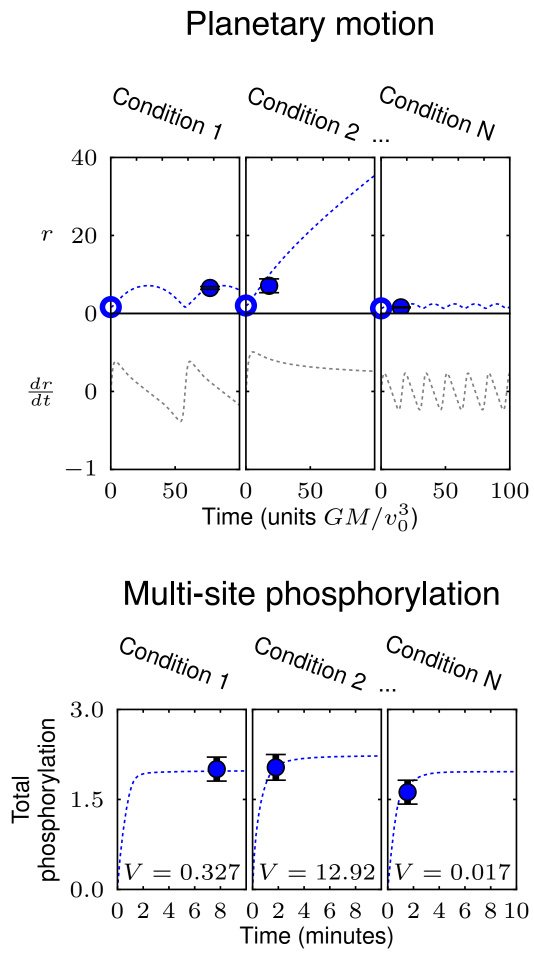

Supplementary Figure 3. Typical in-sample data points for the planetary motion and multi-site phosphorylation model examples. For the planetary motion, r_0 is treated as input, and for each in-sample r_0, r is measured, with added noise, at a single randomly chosen time between 0 and 100. For multi-site phosphorylation, the single parameter V is treated as input, and the total phosphorylation is measured, with added noise, at a single randomly chosen time between 0 and 10 minutes. Dotted lines show the original model behavior, filled circles with error bars show the in-sample data, and unfilled circles show the varying initial conditions in the planetary motion case. The original planetary motion model includes a single hidden variable X_2 corresponding to the time derivative of r. (For the yeast glycolysis example, a similar depiction of typical in-sample data is shown in the left panel of Figure 4.)

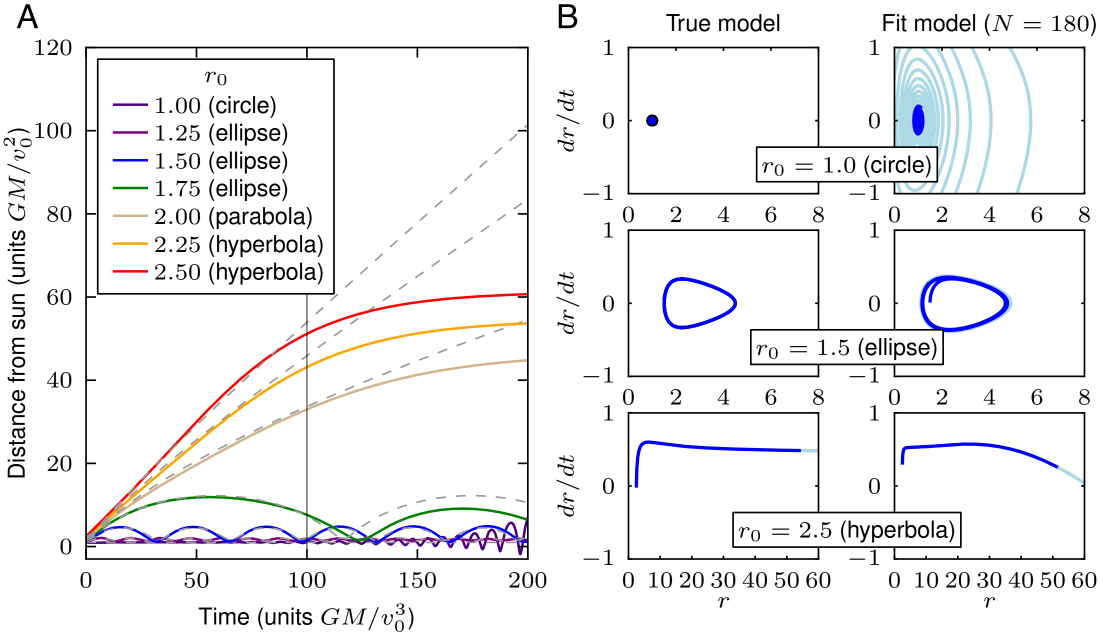

Supplementary Figure 4. Fit of sigmoidal model to planetary data. We know that the sigmoidal network model class is not likely to perform as well for the planetary data case because gravitational interactions do not saturate. Here we show the performance of a model fit to $N = 180$ data points, which contains three hidden variables. The model still fits well in the time region where data is given (between 0 and 100 GM/v_0^3, corresponding to the left half of A and the dark blue part of the trajectories in B), but has a larger divergence from the expected behavior at the extremes of the range of given r_0s in the extrapolated time region (corresponding to the right half of A and the light blue part of the trajectories in B).

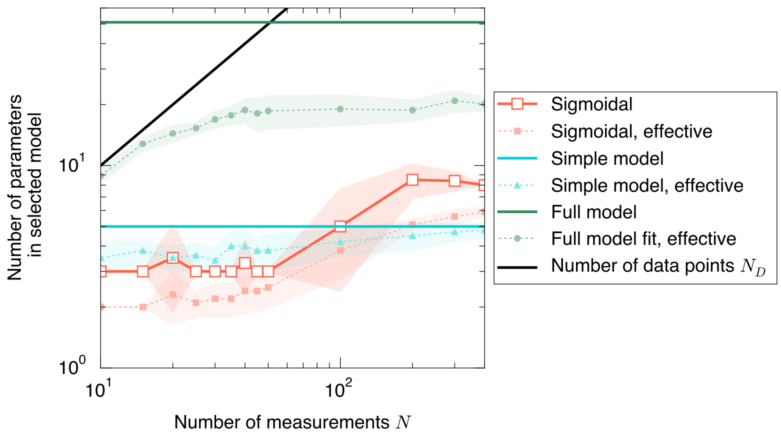

Supplementary Figure 5. Selected adaptive sigmoidal models in the phosphorylation example have both fewer total parameters and fewer effective parameters than the full microscopic model. Solid colored lines indicate the total number of parameters in each model, as in Figure 2 in the main text. Solid symbols connected by dotted lines indicate the effective number of parameters, which we define as the number of directions in parameter space that are constrained by the data such that the corresponding Hessian eigenvalue $\lambda > 1$ (compared to parameter priors with eigenvalue 10^{-2}). Shown are the mean and standard deviation of values over 10 data realizations. For comparison, the solid black line indicates the number of data points $N_{\mathrm{D}} = N$ used to infer the model.

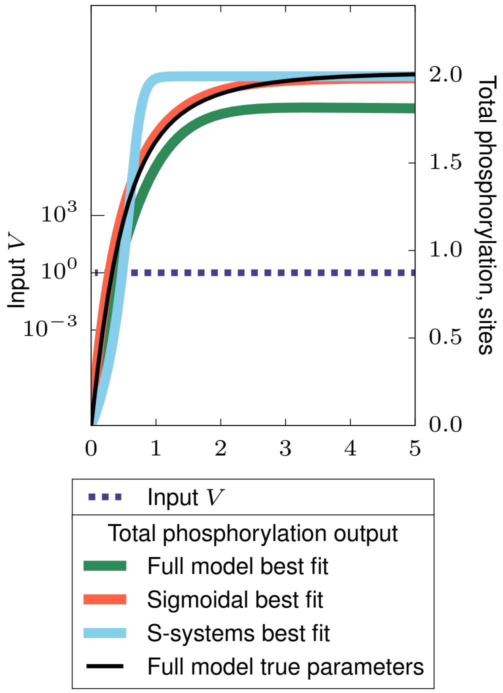

Supplementary Figure 6. A typical example of out-of-sample performance in the multi-site phosphorylation example. Here, each model is fit using $N = 50$ datapoints. With this small amount of data, the differences between model classes are more apparent, with the sigmoidal model class clearly better predicting the dynamics than the S-systems model class and the full phosphorylation model.

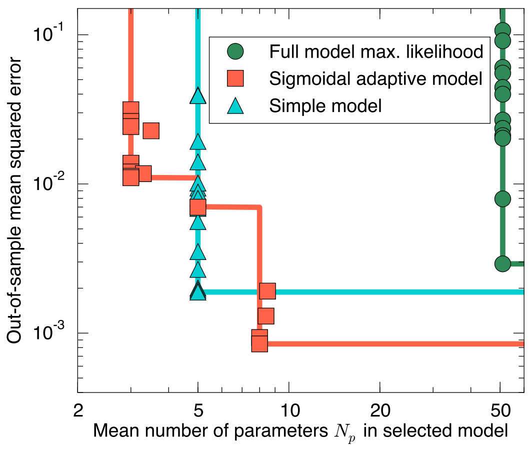

Supplementary Figure 7. The performance of models fit to data from the multi-site phosphorylation model as a function of the number of parameters in each model. This is a replotting of the data in Figure 2 in the main text. If we think of a model as more efficient if it can produce the same level of predictive power with fewer parameters, then the best models lie at the Pareto front, drawn in solid lines for each model type.

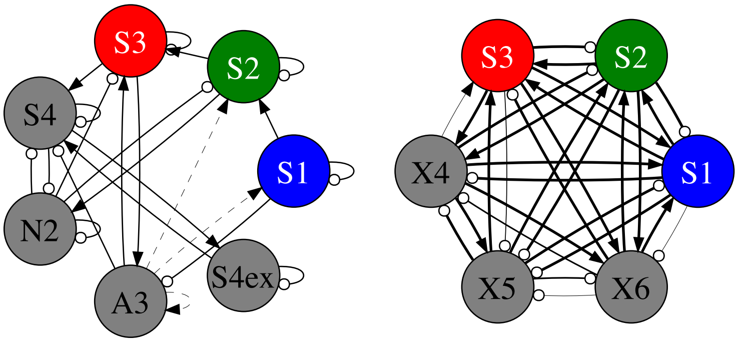

Supplementary Figure 8. (Left) Network depicting the yeast glycolysis model defined by Eqns. (13). Solid arrows represent excitation, solid lines with circles represent inhibition, and dashed arrows represent other types of interaction terms. (Right) Selected sigmoidal network fit to $N = 40$ noisy measurements from the yeast glycolysis model, as shown in Figure 4. Again, arrows represent excitation and circles inhibition, with the thickness of arrows indicating interaction strength. For clarity, self-inhibitory terms for each variable are not shown.

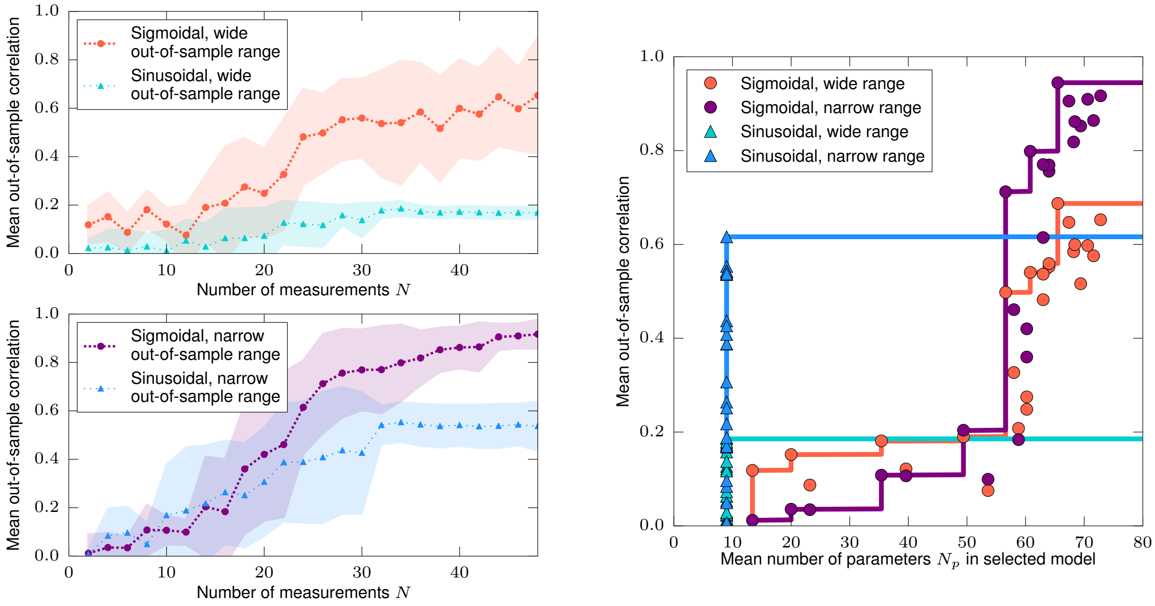

Supplementary Figure 9. Performance of inferred models of yeast glycolysis as a function of the number of measurements N (left) and the mean number of parameters N_p in the selected model (right). The given sigmoidal model hierarchy requires about 30 measurements (corresponding to 90 datapoints) and 60 parameters to produce reasonable predictions. Here we compare mean correlations produced for out-of-sample initial conditions chosen from ranges twice as large as in-sample ranges ("wide ranges," plotted in red, listed in the "out-of-sample" column of Supplementary Table 4) to when out-of-sample conditions are chosen from the same ranges as in-sample ranges ("narrow ranges," plotted in purple, listed in the "in-sample" column of Supplementary Table 4). For comparison, the simple sinusoidal model defined in (15) is shown in shades of blue. The mean and standard deviation over 5 realizations of in-sample data are shown by filled symbols and shaded regions. Also plotted are the Pareto fronts for each model (solid lines on right plot) indicating the maximal correlation for a given mean N_p.

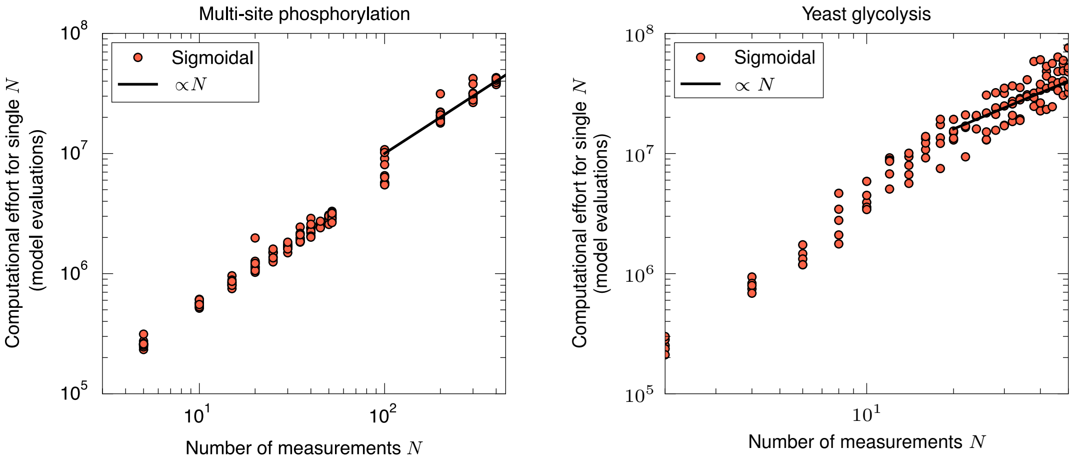

Supplementary Figure 10. The number of model evaluations (integrations) used at each N, for the multi-site phosphorylation and yeast glycolysis examples. Once the size of model has saturated, we expect the number of evaluations to scale linearly with N (black lines). If the selected model size is growing with N, as in the yeast glycolysis example below $N = 20$ (see Supplementary Figure 11), we expect faster than linear growth.

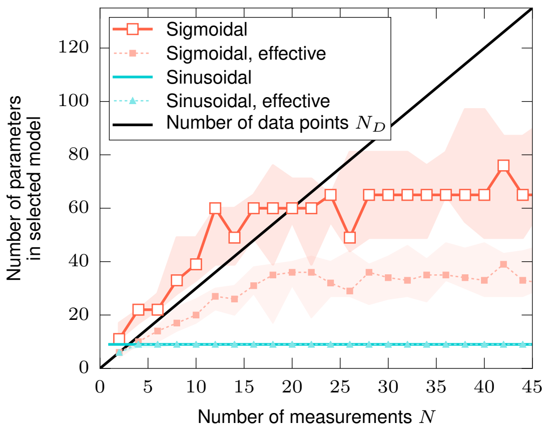

Supplementary Figure 11. Fitting sigmoidal models to the yeast glycolysis oscillation data, the number of total parameters in the selected model, plotted with red open squares, saturates to roughly 65. Plotted with red solid squares is the effective number of parameters, which we define as the number of directions in parameter space that are constrained by the data such that the corresponding Hessian eigenvalue $\lambda > 1$ (compared to parameter priors with eigenvalue 10^{-2}). Corresponding values for the simple sinusoidal model are plotted in blue. Since the blue curve does not grow for $N \geq 5$, we conclude that the simple model does not have the statistical power to fit the data and is too simple for this case. For comparison, the solid black line indicates the number of data points $N_{\mathrm{D}} = 3N$ used to infer the model. We expect the optimal effective number of parameters to stay below N_{D}. Shown are the median and full range of values over 5 data realizations.

Model No. i	Num. parameters N_{p}	Form of power-law ODEs
0	3	$x_1(0) = x_1^{\mathrm{init}}$<br>$\frac{dx_1}{dt} = x_I^{g_{10}} x_1^{g_{11}} - \beta_1$
1	4	$x_1(0) = x_1^{\mathrm{init}}$<br>$\frac{dx_1}{dt} = x_I^{g_{10}} x_1^{g_{11}} - \beta_1 x_I^{h_{10}}$
2	5	$x_1(0) = x_1^{\mathrm{init}}$<br>$\frac{dx_1}{dt} = x_I^{g_{10}} x_1^{g_{11}} - \beta_1 x_I^{h_{10}} x_1^{h_{11}}$
3	6	$x_1(0) = x_1^{\mathrm{init}}$<br>$\frac{dx_1}{dt} = \alpha_1 x_I^{g_{10}} x_1^{g_{11}} - \beta_1 x_I^{h_{10}} x_1^{h_{11}}$
4	8	$x_1(0) = x_1^{\mathrm{init}}$<br>$x_2(0) = x_2^{\mathrm{init}}$<br>$\frac{dx_1}{dt} = \alpha_1 x_I^{g_{10}} x_1^{g_{11}} x_2^{g_{12}} - \beta_1 x_I^{h_{10}} x_1^{h_{11}}$<br>$\frac{dx_2}{dt} = x_2^{g_{22}} - 1$
5	9	$x_1(0) = x_1^{\mathrm{init}}$<br>$x_2(0) = x_2^{\mathrm{init}}$<br>$\frac{dx_1}{dt} = \alpha_1 x_I^{g_{10}} x_1^{g_{11}} x_2^{g_{12}} - \beta_1 x_I^{h_{10}} x_1^{h_{11}} x_2^{h_{12}}$<br>$\frac{dx_2}{dt} = x_2^{g_{22}} - 1$
6	10	$x_1(0) = x_1^{\mathrm{init}}$<br>$x_2(0) = x_2^{\mathrm{init}}$<br>$\frac{dx_1}{dt} = \alpha_1 x_I^{g_{10}} x_1^{g_{11}} x_2^{g_{12}} - \beta_1 x_I^{h_{10}} x_1^{h_{11}} x_2^{h_{12}}$<br>$\frac{dx_2}{dt} = x_1^{g_{21}} x_2^{g_{22}} - 1$

Supplementary Table 1. The first seven models of an example hierarchy in the S-systems class with one input x_I and fixed initial conditions x_1^{init} and x_2^{init}.

Model No. i	Num. parameters N_p	Form of sigmoidal ODEs
0	3	$x_1(0) = x_1^{\mathrm{init}}$ $\frac{dx_1}{dt} = -x_1/\tau_1 + W_{11}\xi(x_1) + W_{10}x_I$
1	4	$x_1(0) = x_1^{\mathrm{init}}$ $\frac{dx_1}{dt} = -x_1/\tau_1 + W_{11}\xi(x_1+\theta_1) + W_{10}x_I$
2	6	$x_1(0) = x_1^{\mathrm{init}}$ $x_2(0) = x_2^{\mathrm{init}}$ $\frac{dx_1}{dt} = -x_1/\tau_1 + W_{11}\xi(x_1+\theta_1) + W_{12}\xi(x_2) + W_{10}x_I$ $\frac{dx_2}{dt} = -x_2$
3	7	$x_1(0) = x_1^{\mathrm{init}}$ $x_2(0) = x_2^{\mathrm{init}}$ $\frac{dx_1}{dt} = -x_1/\tau_1 + W_{11}\xi(x_1+\theta_1) + W_{12}\xi(x_2) + W_{10}x_I$ $\frac{dx_2}{dt} = -x_2 + W_{20}x_I$
4	8	$x_1(0) = x_1^{\mathrm{init}}$ $x_2(0) = x_2^{\mathrm{init}}$ $\frac{dx_1}{dt} = -x_1/\tau_1 + W_{11}\xi(x_1+\theta_1) + W_{12}\xi(x_2) + W_{10}x_I$ $\frac{dx_2}{dt} = -x_2 + W_{21}\xi(x_1+\theta_1) + W_{20}x_I$
5	9	$x_1(0) = x_1^{\mathrm{init}}$ $x_2(0) = x_2^{\mathrm{init}}$ $\frac{dx_1}{dt} = -x_1/\tau_1 + W_{11}\xi(x_1+\theta_1) + W_{12}\xi(x_2) + W_{10}x_I$ $\frac{dx_2}{dt} = -x_2 + W_{22}\xi(x_2) + W_{21}\xi(x_1+\theta_1) + W_{20}x_I$

Supplementary Table 2. The first six models of an example model hierarchy in the sigmoidal class with one input x_I and fixed x_1^{init} and x_2^{init}.

J_0	2.5	mM min^{-1}
k_1	100.	mM^{-1} min^{-1}
k_2	6.	mM^{-1} min^{-1}
k_3	16.	mM^{-1} min^{-1}
k_4	100.	mM^{-1} min^{-1}
k_5	1.28	min^{-1}
k_6	12.	mM^{-1} min^{-1}
k	1.8	min^{-1}
κ	13.	min^{-1}
q	4	
K_1	0.52	mM
ψ	0.1	
N	1.	mM
A	4.	mM

Supplementary Table 3. Parameters for the yeast glycolysis model defined in Eqns. (13).

Variable	In-sample IC (mM)	Out-of-sample IC (mM)	In-sample σ (mM)
S_1	[0.15, 1.60]	[0.15, 3.05]	0.04872
S_2	[0.19, 2.16]	[0.19, 4.13]	0.06263
S_3	[0.04, 0.20]	[0.04, 0.36]	0.00503
S_4	0.115	0.115	N/A
S_5	0.077	0.077	N/A
S_6	2.475	2.475	N/A
S_7	0.077	0.077	N/A

Supplementary Table 4. Initial conditions (IC) and standard deviations of experimental noise (σ) used in the yeast glycolysis model. Initial conditions for visible species S_1, S_2, and S_3 are chosen uniformly from the given ranges, chosen to match Ref. [1]. Out-of-sample ranges are each twice as large as in-sample ranges. Initial conditions for the remaining hidden species are fixed at reference initial conditions from Refs. [1] and [2]. In-sample noise is set at 10% of the standard deviation of each variable's concentration in the limit cycle, as quoted in Ref. [1].

Δp (gravitation and phosphorylation examples)	2
Δp (yeast example)	5
$i_{\text{overshoot}}$	3
Ensemble temperature T (full phosphorylation model)[a]	10
Ensemble temperature T (all other models)	10^3
Total number of Monte Carlo steps (full phosphorylation model)[a]	10^2
Total number of Monte Carlo steps (all other models)	10^4
Number of ensemble members used	10
`avegtol`	10^{-2}
`maxiter`	10^2

Supplementary Table 5. Adaptive inference algorithm parameters. [1]In the full phosphorylation model, we fit parameters in log-space since they are known to be positive. This makes the model more sensitive to large changes in parameters, meaning that we are forced to be more conservative with taking large steps in parameter space to achieve reasonable acceptance ratios.

Supplementary Note 1. HIERARCHICAL BAYESIAN MODEL SELECTION

For consistent inference, we need a hierarchy of models that satisfies criteria laid out in Ref. [3]. First, we desire a model hierarchy that will produce a single maximum in $\mathcal{L}$, up to statistical fluctuations, as we add complexity. For this, the hierarchy should be nested (but not necessarily regular or self-similar), meaning that once a part of the model is added, it is never taken away. Second, the hierarchy should be complete, meaning it is able to fit any data arbitrarily well with a sufficiently complex model. Intuitively, instead of searching a large multidimensional space of models, hierarchical model selection follows a single predefined path through model space (Supplementary Figure 1). While the predefined path may be suboptimal for a particular instance (that is, the true model may not fall on it), even then the completeness guarantees that we will still eventually learn any dynamical system F given enough data, and nestedness assures that this will be done without overfitting along the way.[1]

Ordering of hierarchies: An advantage of the S-systems and sigmoidal representations is the existence of a natural scheme for creating a one-dimensional model hierarchy: simply adding dynamical variables x_i. The most general network is fully connected, such that every variable x_i has an interaction term in every other dx_j/dt. Our hierarchy starts with a fully-connected network consisting of the necessary number of input and output variables, and adds "hidden" dynamical variables to add complexity. With each additional x_i, we add parameters in a predetermined order.

In the S-systems class, without connections, variable x_i's behavior is specified by 5 parameters: $x_i^{\text{init}}, \alpha_i, \beta_i, g_{ii}$, and h_{ii}. Each connection to and from x_j is specified by 4 parameters: g_{ij}, g_{ji}, h_{ij}, and h_{ji}. When adding a new dynamic variable, we first fix its parameters (to zero for the exponential parameters and one for the multiplicative parameters), and then allow them to vary one at a time in the following order: $g_{ii}, g_{ji}, h_{ji}, g_{ij}, h_{ij}, \beta_i, h_{ii}, \alpha_i$ (adding connections to every other x_j one at a time). An example is shown in Supplementary Table 1.

The sigmoidal class is similar: without connections, variable x_i's behavior is specified by 4 parameters: $x_i^{init}, W_{ii}, \tau_i$, and θ_i. Each connection to and from x_j is specified by 2 parameters: W_{ij}

[1] In general, we are not guaranteed good predictive power until $N \to \infty$, but we can hope that the assumptions implicit in our priors (consisting of the specific form of the chosen model hierarchy and the priors on its parameters) will lead to good predictive power even for small N.

and W_{ji}. When adding a new dynamic variable, we first fix its parameters (to zero for W and θ and one for τ), and then allow them to vary one at a time in the following order: $W_{ij}, W_{ji}, W_{ii}, \tau_i, \theta_i$ (adding connections to every other x_j one at a time). An example is shown in Supplementary Table 2.

For every adaptive fit model and the full multi-site phosphorylation model, we use the same prior for every parameter α_k, which we choose as a normal distribution $\mathcal{N}(0, 10^2)$ with mean 0 and standard deviation $\varsigma = 10$.[2] (For the simple model fit to the phosphorylation data, parameters are always well-constrained and priors are unimportant, and we therefore do not use explicit priors.)

Representation of sharp nonlinearities: Both the sigmoidal and S-systems classes can represent arbitrary dynamics. However, it is important that they can *efficiently* represent sharp nonlinearities that are often present in biological systems, such as those typically represented by large Hill coefficients. While this is straightforward for the S-systems class [4], it is less obvious for sigmoidal models.

The sigmoidal model class relies on $\xi(y)$, which has the largest derivative $\xi'(0) = -1$. Thus it may seem that sharp nonlinearities could be hard to produce. In fact, the introduction of hidden variables that perform multiple transformations can produce arbitrarily sharp production rate laws. As an example, we show here that the nonlinearity captured by the Hill equation,

$$f = S^n/(S^n + K), \tag{1}$$

(where S is the substrate concentration, K is the dissociation constant, and f is the fraction of bound receptors) can be represented exactly in the sigmoidal class using two dynamical variables. Treating $I = \log S$ as the input to the system, the sigmoidal system

$$\begin{aligned} \frac{dx_1}{dt} &= -\frac{x_1}{\tau_1} - I, \\ \frac{dx_2}{dt} &= -x_2 + \xi(x_1 + \theta_1), \end{aligned} \tag{2}$$

where we set $\tau_1 = n$ and $\theta_1 = \log K$, has a steady state solution that reproduces (1):

$$\lim_{t\to\infty} x_2(t) = \xi(-n \log S + \log K) = f. \tag{3}$$

[2] Some parameters (α and β in the S-systems model class, τ in the sigmoidal model class, and k and K parameters in the full phosphorylation model) are restricted to be positive, which we accomplish by optimizing over the log of each parameter. The priors are still applied in non-log space, effectively creating a prior that is zero for negative parameter values and $2N(0, 10)$ for positive parameter values.

Robustness of adaptive inference: In Supplementary Figure 2, we test the robustness of the performance of adaptive models in the multi-site phosphorylation example (see below) when various assumptions of the modeling framework are violated.

First, the derivation in Supplementary Note 5 assumes that the distribution of noise on measured data is Gaussian with known variance. In Supplementary Figure 2A, we compare fitting to the same data but using an incorrect standard deviation for noise on the data when calculating the Bayesian log-likelihood. When the data is thought to be noisier than it actually is (purple and red points), performance remains unchanged until large N, when, as expected, simpler than optimal models are chosen, and comparatively more data is required to select complex models that produce better performance. When the data is thought to be less noisy than it actually is (yellow points), more complex models are selected, which in this case yields performance that can be better or worse, depending on N. In Supplementary Figure 2B, we compare fitting to data with log-normally distributed noise, keeping the mean and variance fixed. The closely overlapping performance suggests that, in the absence of knowledge about the true noise distribution, a good estimate of σ may be enough to attain consistent inference.

Finally, a somewhat arbitrary choice must be made to define an ordering for adding parameters in the model hierarchy; we chose to use the "node order" that is described in Supplementary Table 2. In Supplementary Figure 2C, we instead add parameters for each dynamical variable in random order. This includes orderings that first add parameters controlling only hidden nodes, which may be decoupled from the visible variables and hence cannot improve the fit. To compensate for this and avoid erroneously stopping fitting due to adding these unproductive parameters, we increase the number of models checked by increasing $i_{\text{overshoot}}$ from 3 to 4 (see Supplementary Note 6). One could additionally avoid unproductive orderings by checking that each additional parameter has some causal influence on visible variables. But even including these orderings, mean performance is largely unaffected.

Supplementary Note 2. THE LAW OF GRAVITY MODEL

For a mass m in motion under the influence of the gravitational field of a mass $M \gg m$, the distance r between the two evolves as [5]

$$\frac{d^2r}{dt^2} = \frac{h^2}{r^3} - \frac{GM}{r^2}, \tag{4}$$

where $h = (\mathbf{v}_0 \cdot \hat{\theta})r_0$ is the specific angular momentum, $\mathbf{v}_0$ is the initial velocity, r_0 is the initial distance, $\hat{\theta}$ is the unit vector perpendicular to the line connecting the two masses, and G is the gravitational constant. Setting the initial velocity parallel to $\hat{\theta}$ and measuring distance in units of $\frac{GM}{v_0^2}$ and time in units of $\frac{GM}{v_0^3}$, the dynamics become[3]

$$\frac{d^2r}{dt^2} = \frac{1}{r^2}\left(\frac{r_0^2}{r} - 1\right). \tag{5}$$

When written as two first-order differential equations, we see that this system can be represented exactly in the S-systems class if the particle does not fall onto the Sun:

$$\begin{aligned} \frac{dr}{dt} &= \chi - 1 \\ \frac{d\chi}{dt} &= r_0^2 r^{-3} - r^{-2}, \end{aligned} \tag{6}$$

where we use the variable $\chi = \frac{dr}{dt} + 1$, so that the resulting system's variables are never negative, a requirement of the S-systems class.

To illustrate constructing an adaptive model for planetary motion, we consider as input the initial distance from the sun r_0. We sample r_0 uniformly between 1 and 3 (in units of GM/v_0^2), which covers the possible types of dynamics: at $r_0 = 1$, the orbit is circular; when $1 < r_0 < 2$ the orbit is elliptical; when $r_0 = 2$ the orbit is parabolic; and when $r_0 > 2$ the orbit is hyperbolic. In this and later examples, to best determine the minimum number of measurements needed for a given level of performance, we sample the system at a single time point for each initial condition (Supplementary Figure 3), rather than sampling a whole trajectory per condition. This ensures that samples are independent, which would not be the case for subsequent data points of the same trajectory, and hence allows us to estimate the data requirements of the algorithm more

[3] Note that r_0 sets the (conserved) angular momentum: $h = \frac{GM}{v_0} r_0$ with r_0 in rescaled units.

reliably. Further, this is similar to the sampling procedure already used in the literature [1]. In the planetary motion case, we assume only the distance r is measured, meaning the total number of of datapoints $N_{\rm D} = N$, where N is the number of initial conditions sampled. We choose the time of the observation as a random time uniformly chosen between 0 and 100, with time measured in units of GM/v_0^3. To each measurement we add Gaussian noise with standard deviation equal to 5% of the maximum value of r between $t = 0$ and $t = 100\ GM/v_0^3$.

Typical training data for the model can be seen in Supplementary Figure 3. Fits to $N = 150$ data points are shown in Figure 1. Here our adaptive fitting algorithm selects a model of the correct dimension, with one hidden variable. The selected model ODEs in this case are

$$\begin{aligned}\frac{dr}{dt} &= e^{-3.405} r_0^{3.428} r^{0.049} X_2^{7.372} - e^{-2.980} r_0^{2.936} r^{0.046} X_2 {-4.925} \\ \frac{dX_2}{dt} &= r_0^{-0.651} r^{-3.435} X_2^{-0.014} - e^{-0.006} r_0^{-4.288} r^{-1.595}.\end{aligned} \tag{7}$$

Note that certain transformations of the hidden variable and parameters can leave the output behavior unchanged while remaining in the S-systems class. First, the initial condition of hidden parameters can be rescaled to 1 without loss of generality, so we remove this degree of freedom and set $X_2(0) = 1$. Second, we have the freedom to let the hidden variable $X_2 \to X_2^\gamma$ for any $\gamma \neq 0$ with appropriate shifts in parameters. To more easily compare the fit model with the perfect model, in the rightmost column of Figure 1we plot X_2^2 on the vertical axes instead of X_2 when comparing it to the dynamics of the true hidden variable χ.

Finally, we may compare performance when we fit the gravitation data using sigmoidal models, a model class that we know is not representative of the underlying mechanics. The results are shown in Supplementary Figure 4; the selected sigmoidal network, which contains three hidden variables, still provides a good fit to the data, as expected, but it does not generalize as well when r_0 is near the edge of the range contained in the data and timepoints are outside of the range of data to which they were fit. This is expected since forces can diverge in the true law of gravity, and they are necessarily limited in the sigmoidal model.

Supplementary Note 3. MULTI-SITE PHOSPHORYLATION MODEL

To explore a complicated biological system with relatively simple output behavior, we imagine a situation in which an immune receptor can be phosphorylated at each of five sites arranged in a linear chain. The rates of phosphorylation and dephosphorylation at each site are affected by the phosphorylation states of its nearest neighboring sites. A site can be unphosphorylated (U) or phosphorylated (P), and its state can change via one of two processes. The first process does not depend on states of neighboring sites:

$$U_i \rightleftharpoons P_i, \tag{8}$$

with on-rate $k_i^{\text{on}}([U_i])$ and off-rate $k_i^{\text{off}}([P_i])$ that depend on the concentration of the corresponding substrate. The second, cooperative process happens only when a neighboring site j is phosphorylated:

$$U_i P_j \rightleftharpoons P_i P_j \tag{9}$$

with on- and off-rates $k_{ij}^{\text{on}}([U_i P_j])$ and $k_{ij}^{\text{off}}([P_i P_j])$. All rates k are modeled as Michaelis-Menten reactions: $k([S]) = \frac{V[S]}{K_{\text{m}}+[S]}$. With each reaction specified by two parameters (V and K_{m}) and 26 possible reactions, the phosphorylation model has a total of 52 parameters. To more easily generate the differential equations that govern the multi-site phosphorylation model, we use the BioNetGen package [6, 7].

When fitting this phosphorylation model, we use as input the parameter V_{23}^{on}, which is chosen from a uniform distribution in log-space between 10^{-3} and 10^{3} min^{-1}. The remaining 51 V and K_{m} parameters we sample randomly from our priors on these parameters. As output, we measure the total phosphorylation of the 5 sites P_{tot} at a single random time uniformly chosen between 0 and 10 minutes. To each measurement we add Gaussian noise with standard deviation equal to 10% of the P_{tot} value at $t = 10$ min.

Typical training data for the model is shown in Supplementary Figure 3. The out-of-sample mean squared error, as plotted in Figure 2, is measured over 100 new input values selected from the same distribution as the in-sample values, each of which is compared to the true model at 100

timepoints evenly spaced from 0 to 10 minutes.

As a simple guess to the functional form of the total phosphorylation timecourse as a function of our control parameter $V = V_{23}^{\text{on}}$ (the "simple model" in Figure 2), we use an exponential saturation starting at 0 and ending at a value P_∞ that depends sigmoidally on V:

$$P_{\text{tot}} = P_\infty(V)\left[1 - \exp\left(\frac{t}{t_0}\right)\right], \tag{10}$$

where

$$P_\infty(V) = a + \frac{b}{2}\left[1 + \tanh\left(\frac{\log(V) - d}{c}\right)\right] \tag{11}$$

and a, b, c, d, and t_0 are parameters fit to the data. Figure 2 shows that this simple *ad hoc* model can fit the data quite well.

For the example shown in Figure 3, the selected sigmoidal model consists of the ODEs

$$\begin{aligned}
\frac{dP_{\text{tot}}}{dt} &= \frac{-P_{\text{tot}}}{e^{-1.219}} + \frac{0.409}{1 + \exp(P_{\text{tot}} - 4.469)} + \frac{7.087}{1 + \exp(X_2)} + 0.0005V \\
\frac{dX_2}{dt} &= -X_2 - \frac{2.303}{1 + \exp(P_{\text{tot}} - 4.469)} - 0.071V \\
X_2(0) &= 0.101,
\end{aligned} \tag{12}$$

with $P_{\text{tot}}(0) = 0$.

The selected sigmoidal models contain fewer parameters than the microscopic exact model, even when taking into account that the full model is effectively lower dimensional, with many directions in parameter space unconstrained by typical data; see Supplementary Figure 5.

In this multi-site phosphorylation example, the sigmoidal model class is a better performer than the S-systems class. A typical example of performance is depicted in Supplementary Figure 6. Though the S-systems class makes predictions that are still qualitatively correct, and its predictions steadily improve as N increases, the sigmoidal class comes closer to the true underlying model with an equal amount of data.

The confidence intervals on the dynamics in Figure 3 correspond to samples from the posterior over parameters given $N = 300$ data points. In the notation of Supplementary Note 5, this posterior $P(\alpha \mid \text{data}) \propto \exp\left[-\tilde{\chi}^2(\alpha)/2\right]$. To generate samples from this distribution, we use

Metropolis Monte Carlo as implemented in SloppyCell [8, 9]. As a starting point, we use the best-fit parameters from the model selection procedure, and we sample candidate steps in parameter space from a multidimensional Gaussian corresponding to the Hessian at the best-fit parameters.[4] From 10^4 Monte Carlo steps, the first half are removed to avoid bias from the initial condition, and every 50 of the remaining steps are used as 100 approximately independent samples from the parameter posterior.

Supplementary Figure 7 replots the performance data from Figure 2 as a function of the number of parameters in each model, showing a Pareto front indicating the minimum number of parameters needed to reach a given level of performance using these models.

Supplementary Note 4. YEAST GLYCOLYSIS MODEL

As an example of inference of more complicated dynamics, we use a model of oscillations in yeast glycolysis, originally studied in terms of temperature compensation [2] and since used as a test system for automated inference [1]. The model's behavior is defined by ODEs describing the dynamics of the concentrations of seven molecular species (the biological meaning of the species is not important here):

$$\begin{aligned}
\frac{dS_1}{dt} &= J_0 - \frac{k_1 S_1 S_6}{1 + (S_6/K_1)^q} \\
\frac{dS_2}{dt} &= 2\frac{k_1 S_1 S_6}{1 + (S_6/K_1)^q} - k_2 S_2 (N - S_5) - k_6 S_2 S_5 \\
\frac{dS_3}{dt} &= k_2 S_2 (N - S_5) - k_3 S_3 (A - S_6) \\
\frac{dS_4}{dt} &= k_3 S_3 (A - S_6) - k_4 S_4 S_5 - \kappa (S_4 - S_7) \\
\frac{dS_5}{dt} &= k_2 S_2 (N - S_5) - k_4 S_4 S_5 - k_6 S_2 S_5 \\
\frac{dS_6}{dt} &= -2\frac{k_1 S_1 S_6}{1 + (S_6/K_1)^q} + 2 k_3 S_3 (A - S_6) - k_5 S_6 \\
\frac{dS_7}{dt} &= \psi \kappa (S_4 - S_7) - k S_7 .
\end{aligned} \tag{13}$$

Parameter values, listed in Supplementary Table 3, are set to match with those used in Ref. [1] and Table 1 of Ref. [2], where our $S_5 = N_2$, our $S_6 = A_3$, and our $S_7 = S_4^{\text{ex}}$.

[4] Unconstrained parameter directions in the proposal distribution, corresponding to singular values smaller than $\lambda_{\text{cut}} = \lambda_{\max}/10$, where $\lambda_{\max}$ is the largest singular value, are cut off to λ_{cut} to produce reasonable acceptance ratios (near 0.5).

For the yeast glycolysis model, we use as input the initial conditions for the visible species S_1, S_2, and S_3. These are each chosen uniformly from ranges listed in the "In-sample IC" column of Supplementary Table 4. Each of the three visible species are then measured at a random time uniformly chosen from 0 to 5 minutes, meaning the total number of datapoints $N_\mathrm{D} = 3N$ for this system, where N is the number of initial conditions sampled. Gaussian noise is added to each measurement with standard deviations given in Supplementary Table 4. To evaluate the model's performance, we test it using 100 new input values selected uniformly from the ranges listed in the "Out-of-sample IC" column of Supplementary Table 4, each of which is compared to the true model at 100 timepoints evenly spaced from 0 to 5 min. The correlation between the adaptive fit model and the actual model over these 100 timepoints is calculated separately for each visible species, set of initial conditions, and in-sample data, and the average is plotted as the "mean out-of-sample correlation" in Figure 4. The topology of the selected sigmoidal model in an example with $N = 40$

is illustrated in Supplementary Figure 8. The model ODEs in this case are

$$
\begin{aligned}
\frac{dS_1}{dt} &= \frac{-S_1}{e^{2.284}} + \frac{2.520}{1+\exp(S_1-0.4246)} + \frac{14.04}{1+\exp(S_2-0.4943)} - \frac{19.56}{1+\exp(S_3+0.6711)} \\
&\quad - \frac{10.68}{1+\exp(X_4+2.240)} + \frac{6.759}{1+\exp(X_5-0.7566)} - \frac{3.051}{1+\exp(X_6)} \\
\frac{dS_2}{dt} &= \frac{-S_2}{e^{-1.288}} - \frac{3.015}{1+\exp(S_1-0.4246)} + \frac{2.244}{1+\exp(S_2-0.4943)} + \frac{14.55}{1+\exp(S_3+0.6711)} \\
&\quad + \frac{25.77}{1+\exp(X_4+2.240)} - \frac{6.699}{1+\exp(X_5-0.7566)} - \frac{4.380}{1+\exp(X_6)} \\
\frac{dS_3}{dt} &= \frac{-S_3}{e^{1.514}} - \frac{2.463}{1+\exp(S_1-0.4246)} - \frac{10.99}{1+\exp(S_2-0.4943)} + \frac{0.6530}{1+\exp(S_3+0.6711)} \\
&\quad - \frac{0.07038}{1+\exp(X_4+2.240)} - \frac{6.806}{1+\exp(X_5-0.7566)} + \frac{12.61}{1+\exp(X_6)} \\
\frac{dX_4}{dt} &= \frac{-X_4}{e^{1.771}} + \frac{25.77}{1+\exp(S_1-0.4246)} - \frac{50.05}{1+\exp(S_2-0.4943)} - \frac{6.648}{1+\exp(S_3+0.6711)} \\
&\quad - \frac{59.44}{1+\exp(X_4+2.240)} + \frac{52.34}{1+\exp(X_5-0.7566)} + \frac{1.148}{1+\exp(X_6)} \\
\frac{dX_5}{dt} &= \frac{-X_5}{e^{-2.513}} + \frac{16.39}{1+\exp(S_1-0.4246)} + \frac{33.15}{1+\exp(S_2-0.4943)} + \frac{0.6452}{1+\exp(S_3+0.6711)} \\
&\quad - \frac{33.65}{1+\exp(X_4+2.240)} - \frac{8.976}{1+\exp(X_5-0.7566)} + \frac{0.01966}{1+\exp(X_6)} \\
\frac{dX_6}{dt} &= -X_6 + \frac{0.3391}{1+\exp(S_1-0.4246)} - \frac{2.514}{1+\exp(S_2-0.4943)} - \frac{4.479}{1+\exp(S_3+0.6711)} \\
&\quad - \frac{3.396}{1+\exp(X_4+2.240)} + \frac{1.219}{1+\exp(X_5-0.7566)} + \frac{2.313}{1+\exp(X_6)} \\
X_4(0) &= 3.437 \\
X_5(0) &= 1.453 \\
X_6(0) &= -0.7183.
\end{aligned}
\tag{14}
$$

Note that our model fitting approach assumes that the model timecourse is fully determined (aside from measurement error) by the concentrations of measured species. To be consistent with this assumption we do not vary the initial conditions of the three hidden variables. In future work it may be possible to relax this assumption, allowing the current state of intrinsic variations in hidden variables to be learned as well.

Simple sinusoidal model: As with the multi-state phosphorylation example, we can use a simple *ad hoc* model of yeast glycolysis for comparison to our adaptive models. The long-term behavior of the yeast network consists of stable oscillations with a roughly fixed period; a minimally complicated model of the measured concentrations S_1, S_2, and S_3 then consists of three sinusoidal

oscillators with equal frequency ω and phase relationship fixed by two parameters, ϕ_2 and ϕ_3:

$$\begin{aligned} S_1(t) &= y_1 + A_1 \sin(\omega t + \phi) \\ S_2(t) &= y_2 + A_2 \sin(\omega t + \phi + \phi_2) \\ S_3(t) &= y_3 + A_3 \sin(\omega t + \phi + \phi_3). \end{aligned} \tag{15}$$

The phase ϕ depends on the initial conditions $S_1(0), S_2(0), S_3(0)$. Specifically, when the initial condition is a valid point on the one-dimensional elliptical curve specified by Eqs. (15), ϕ can be determined by any two initial values; for instance,

$$\phi = \arctan \frac{x_1 \sin(\phi_2)}{x_2 - x_1 \cos(\phi_2)}, \tag{16}$$

where $x_i = (S_i(0) - y_i)/A_i$. Because the model is not exact, however, we cannot assume that initial conditions will lie on this curve. Instead, we will assume that transient dynamics infinitely quickly bring the state of the system into the plane defined by the curve. This plane has normal vector $\mathbf{n} = (\sin(\phi_2 - \phi_3), \sin\phi_3, -\sin\phi_2)$, so that any initial conditions $\mathbf{x}$ can be projected onto a point on the plane $\mathbf{x}' = \mathbf{x} - c\mathbf{n}$, where $c = (\mathbf{x} \cdot \mathbf{n})/(\mathbf{n} \cdot \mathbf{n}) = (x_1 \sin(\phi_2 - \phi_3) + x_2 \sin\phi_3 - x_3 \sin\phi_2)/(\sin^2(\phi_2 - \phi_3) + \sin^2\phi_2 + \sin^2\phi_3)$. Thus $\mathbf{x}'$ is a modified initial condition that is inserted into (16) to obtain ϕ. Unlike the adaptive model, this simple sinusoidal model does not capture the jagged shape of the yeast glycolysis oscillations, but when its 9 parameters are fit to data, its rough approximation is moderately predictive. Its performance is compared to sigmoidal adaptive models in Supplementary Figure 9.

Comparing to EUREQa: In Ref. [1], the EUREQa engine is used to infer the same yeast glycolysis model that we use here. We can roughly compare performance as a function of computational and experimental effort by measuring the number of required model evaluations and measurements (Figure 4). Here we compare the two approaches in more detail. However, we emphasize that they have different goals: EUREQa aims at finding the exact microscopic model of the process, while Sir Isaac strives for accurate prediction with the simplest phenomenological model. The former is a harder task, and thus one expects it to require more data and computation.

Reference [1] attempts to match time derivatives of species concentrations as a function of species concentrations, instead of species concentrations as a function of time as we do. This

means that each model evaluation[5] is more computationally costly for us, since it requires an integration of the ODEs over time. It also means, however, that we are able to match well the phases of oscillations, which remain unconstrained in Ref. [1]. The fitting of time courses instead of derivatives also makes our method focus on the fitting of dynamics near the attractor, rather than attempting to constrain dynamics through the entire phase space.

To consistently infer exact equations for the full 7-dimensional model, Ref. [1] used $20,000$ datapoints and roughly 10^{11} model evaluations. We contrast this with our method that produces reasonable inferred models using 40 datapoints and less than 5×10^8 model evaluations (Figure 4).

Finally, in the main text we test the performance of our yeast glycolysis models for out-of-sample ranges of initial conditions that are twice as large as the in-sample ranges from which data is taken, as in Ref. [1], in order to more directly test their ability to extrapolate to regimes that were not tested in training. In Supplementary Figure 9, we compare this to performance when out-of-sample initial conditions are chosen from the same ranges as in-sample data (note that, nonetheless, none of the test examples has appeared in the training set). Here we see that the mean correlation can reach 0.9 using $N = 40$ measurements.

Comparing to dynamical inference with alternation regression: In Ref. [10], we used a somewhat similar dynamical inference algorithm for analysis of the same glycolysis model. It is worthwhile contrasting the two methods.

First, as we have argued here, the crucial difficulty of adaptive dynamical inference is to account for both arbitrary nonlinearities and an arbitrary number of hidden variables underlying data. Sir Isaac does this by traversing hierarchies of models that are complete and can deal with any number of missing variables (e. g., four in the glycolysis example). In contrast, Ref. [10] required knowing every variable in the system, similarly to the EUREQa implementation discussed above [1]. While Ref. [10] controlled the number of (nonlinear) interactions in the system adaptively using Bayesian model selection, similarly to Sir Isaac, there new variables could not be added. Thus the maximum complexity of the model was limited, the model hierarchy was not complete, and the process was not guaranteed to find accurate solutions even with an infinite amount of data. Second, the aim of Sir

[5] In our setup, we define a model evaluation as a single integration of the model ODEs (see Supplementary Note 7).

Isaac is to predict the temporal dynamics of the studied system by inferring the dynamical system from time series and by integrating the inferred dynamics with new initial conditions. In contrast, in Ref. [10], we followed the same route as Ref. [1] and required knowing derivatives in addition to values of variables. Further, the aim of the approach was to predict the derivatives, but not the variables themselves. In particular, there were no guarantees that the integrated trajectories would be close to the true ones, or even that they would stay within a physically reasonable range (such as positive concentrations).

The strong limitations of Ref. [10] resulted in a much smaller computational complexity when the S-systems model hierarchy was used. Indeed, just like Sir Isaac, that method was able to reach out-of sample correlations of 0.6 or higher (for derivatives, rather than the variables themselves) after only a few dozens of training samples. (Parenthetically, we note that the performance saturated then, in agreement with the understanding that the model hierarchy was not complete.) However, the computational time was nearly negligible. It required only a handful of evaluations of linear regressions to infer the model, in contrast to Sir Isaac, which performs a non-convex optimization at each model fitting step. Whether such computational speedup is worth the additional limitations imposed by the algorithm of Ref. [10] will depend on the problem being solved.

Supplementary Note 5. DERIVATION OF BAYESIAN LOG-LIKELIHOOD ESTIMATE $\mathcal{L}$

Multiple previous approaches have used approximate sampling methods to perform Bayesian model selection on a small number of alternate models in the context of systems biology; e. g., [11–13]. For our approach that relies on a search over an infinite set of models, even such approximate sampling is slow. Yet with sufficiently large N, an expansion resembling that used to derive the Bayesian Information Criterion produces good performance without sampling. The derivation here largely follows Refs. [14, 15], but can be traced to the 1970s [16].

For a given model M that depends on parameters α, our model selection algorithm requires an estimate of the probability that M is the model that produced a given set of data $\{y_i\}$ with corresponding error estimates $\{\sigma_i\}$ (measured at a set of timepoints $\{t_i\}$), and $i = 1, \ldots, N$, so that there are N measurements. Since the parameters α are unknown aside from a prior distribution

$P(\alpha)$, we must integrate over all possible values:

$$P(M \mid \text{data}) = P(M \mid \{y_i, \sigma_i, t_i\}) \tag{17}$$

$$= Z_\alpha^{-1} \int d^{N_\text{p}}\alpha \; P(M \mid \{y_i, \sigma_i, t_i\}; \alpha) \; P(\alpha), \tag{18}$$

where the normalization constant $Z_\alpha = \int d^{N_\text{p}}\alpha \; P(\alpha)$ and N_p is the number of parameters. In terms of the output given the model, Bayes rule states

$$P\left(M \mid \{y_i, \sigma_i, t_i\}; \alpha\right) = \frac{P\left(M\right)}{P\left(\{y_i\}\right)} P\left(\{y_i\} \mid M(\alpha); \{\sigma_i, t_i\}\right). \tag{19}$$

Assuming that the model output has normally distributed measurement errors,

$$\begin{aligned} P\left(\{y_i\} \mid M(\alpha); \{\sigma_i, t_i\}\right) &= \prod_{i=1}^{N} P\left(y_i \mid M(\alpha); \sigma_i; t_i\right) \\ &= \prod_{i=1}^{N} \frac{1}{\sqrt{2\pi\sigma_i^2}} \exp\left[-\frac{1}{2}\left(\frac{y_i - M(t_i, \alpha)}{\sigma_i}\right)^2\right] \\ &= Z_\sigma^{-1} \exp\left[-\frac{1}{2}\sum_{i=1}^{N}\left(\frac{y_i - M(t_i, \alpha)}{\sigma_i}\right)^2\right] \\ &= Z_\sigma^{-1} \exp\left[-\frac{1}{2}\chi^2(M(\alpha), \{y_i, \sigma_i, t_i\})\right], \end{aligned} \tag{20}$$

where χ^2 is the usual goodness-of-fit measure consisting of the sum of squared residuals, and Z_σ is the normalization constant $\prod_{i=1}^{N} \sqrt{2\pi\sigma_i^2}$. Thus we have:[6]

$$P(M \mid \text{data}) = C Z_\alpha^{-1} \int d^{N_\text{p}}\alpha \quad \exp\left[-\frac{1}{2}\tilde{\chi}^2(\alpha)\right], \tag{21}$$

where $C \equiv 2P(M)/Z_\sigma P\left(\{y_i\}\right)$ and $\tilde{\chi}^2(\alpha) = \chi^2(\alpha) - 2\log P(\alpha)$. Since we will be comparing models fitting the same data, and we assume all models have the same prior probability $P(M)$, C will be assumed constant in all further comparisons (but see Ref. [17] for the discussion of this assumption).

If there are enough data to sufficiently constrain the parameters (as is the case for ideal data in the limit $N \to \infty$), then the integral will be dominated by the parameters near the single set of best-fit parameters α_best. To lowest order in $1/N$, we can approximate the integral using a saddle-point approximation [15]:

$$P(M \mid \text{data}) \approx C Z_\alpha^{-1} \exp\left[-\frac{1}{2}\tilde{\chi}^2(\alpha_\text{best})\right] \int d^{N_\text{p}}\alpha \; \exp\left[-(\alpha - \alpha_\text{best})\mathcal{H}(\alpha - \alpha_\text{best})\right], \tag{22}$$

[6] We simplify notation by letting $\chi^2(\alpha) = \chi^2(M(\alpha), \{y_i, \sigma_i, t_i\})$.

where $\mathcal{H}$ is the Hessian:[7]

$$\mathcal{H}_{k\ell} = \frac{1}{2}\frac{\partial^2 \tilde{\chi}^2(\alpha)}{\partial\alpha_k d\alpha_\ell}\bigg|_{\alpha_{\text{best}}}. \tag{23}$$

If we assume normally distributed priors on parameters with variances ς_k^2, the log posterior probability becomes

$$\log P(M \mid \text{data}) \approx \text{const} - \frac{1}{2}\tilde{\chi}^2(\alpha_{\text{best}}) - \frac{1}{2}\sum_{\mu=1}^{N_{\text{p}}}\log\lambda_\mu - \frac{1}{2}\sum_{k=1}^{N_{\text{p}}}\log\varsigma_k^2, \tag{24}$$

where λ_μ are the eigenvalues of $\mathcal{H}$, and the last term comes from Z_α. We thus use as our measure of model quality

$$\mathcal{L} \equiv -\frac{1}{2}\tilde{\chi}^2(\alpha_{\text{best}}) - \frac{1}{2}\sum_{\mu}\log\lambda_\mu - \frac{1}{2}\sum_{k}\log\varsigma_k^2. \tag{25}$$

Eq. (25) is a generalization of the Bayesian Information Criterion (BIC) [16] when parameter sensitivities and priors are explicitly included.[8] The first term is the familiar χ^2 "goodness of fit," and the last two terms constitute the fluctuation "penalty" for overfitting or complexity. Note that here the goodness of fit and the complexity penalty are both functions of the entire dynamics, rather than individual samples, which is not a common application of Bayesian model selection techniques.

Supplementary Note 6. FITTING ALGORITHM

We are given N data points $\mathbf{x}_i$ at known times t_i and known exogenous parameters I_i, and with known or estimated variances σ_i^2. We are approximating the functions $\mathbf{F}_x$ and $\mathbf{F}_y$ in Eq. (1), where $\mathbf{y}$ are hidden dynamic model variables, and $\mathbf{x} = \mathbf{x}(t, \mathbf{I})$ and $\mathbf{y} = \mathbf{y}(t, \mathbf{I})$ in general depend on time t and inputs $\mathbf{I}$. As described in Supplementary Note 5, we fit to the data $\mathbf{x}_i$ using a combination of squared residuals from the data and priors $P(\alpha)$ on parameters α, which we assume to be Gaussian and centered at zero:

$$\tilde{\chi}^2 = \sum_{i=1}^{N}\left(\frac{\mathbf{x}_i - \mathbf{x}(t_i, I_i)}{\sigma_i}\right)^2 + 2\sum_{k=1}^{N_{\text{p}}}\left(\frac{\alpha_k}{\varsigma_k}\right)^2, \tag{26}$$

---

[7] Near the best-fit parameters where residuals are small, and when priors are Gaussian, $\mathcal{H}$ is approximated by the Fisher Information Matrix, which depends only on first derivatives of model behavior: $\mathcal{H} \approx J^{\text{T}}J + \Sigma^{-2}$, where the Jacobian $J_{i\ell} = \frac{1}{\sigma_i}\frac{\partial M_i}{\partial\alpha_\ell}$ and the diagonal matrix $\Sigma_{k\ell}^{-2} = \delta_{k\ell}\varsigma_k^{-2}$ expresses the effects of parameter priors.

[8] For well-constrained parameters, we expect, to lowest order in $1/N$, our result to be equal to the BIC result of $-\frac{1}{2}\tilde{\chi}^2(\alpha_{\text{best}}) + \frac{1}{2}N_{\text{p}}\log N$.

where F's are integrated to produce the model values $\mathbf{x}$ and $\mathbf{y}$:

$$\mathbf{x}(t, \mathbf{I}) = \mathbf{x}_0(\mathbf{I}) + \int_0^t \mathbf{F}_x(\mathbf{x}(s, \mathbf{I}), \mathbf{y}(s, \mathbf{I}))\ ds \tag{27}$$

$$\mathbf{y}(t, \mathbf{I}) = \mathbf{y}_0(\mathbf{I}) + \int_0^t \mathbf{F}_y(\mathbf{x}(s, \mathbf{I}), \mathbf{y}(s, \mathbf{I}))\ ds. \tag{28}$$

To fit parameters, we use a two step process akin to simulated annealing that uses samples from a "high temperature" Monte Carlo ensemble as the starting points for local optimization performed using a Levenberg-Marquardt routine. The phenomenological models are implemented using SloppyCell [8, 9] in order to make use of its parameter estimation and sampling routines.

Following is a high-level description of the fitting algorithm, with choices of parameters for the examples in the main text listed in Supplementary Table 5.

1. Choose a model class, consisting of a sequence of nested models indexed by i, where the number of parameters N_p monotonically increases with i. Choose a step size Δp.

2. Given data at N_total timepoints, fit to data from the first N timepoints, where N is increased to N_total in steps of ΔN.

3. At each N, test models of increasing number of parameters N_p (stepping by Δp) until $\mathcal{L}$ consistently decreases (stopping when the last $i_\mathrm{overshoot}$ models tested have smaller $\mathcal{L}$ than the maximum). For each model, to calculate $\mathcal{L}$:

   (a) Generate an ensemble of starting points in parameter space using Metropolis-Hastings Monte Carlo to sample from $P(\alpha) \propto \exp(-\tilde{\chi}^2(\alpha)/2TN_\mathrm{D})$ with $\tilde{\chi}^2$ from (26). The temperature T is set large to encourage exploration of large regions of parameter space, but if set too large can result in a small acceptance ratio. Infinities and other integration errors are treated as $\tilde{\chi}^2 = \infty$.

      i. Use as a starting point the best-fit parameters from a smaller N_p if a smaller model has been previously fit, or else default parameters.

      ii. As a proposal distribution for candidate steps in parameter space, use an isotropic Gaussian with standard deviation $\sqrt{TN_\mathrm{D}}/\lambda_\mathrm{max}$, where N_D is the total number of

data residuals and $\lambda_{\max}$ is the largest singular value of the Hessian [Eq. (23)] at the starting parameters.

iii. If this model has previously been fit to less data, use those parameters as an additional member of the ensemble.

(b) Starting from each member of the ensemble, perform a local parameter fit, using Levenberg-Marquardt to minimize $\tilde{\chi}^2$ from (26). Stop when convergence is detected (when the L1 norm of the gradient per parameter is less than `avegtol`) or when the number of minimization steps reaches `maxiter`. The best-fit parameters α^* are taken from the member of the ensemble with the smallest resulting fitted $\tilde{\chi}^2$.

(c) At α^*, calculate $\mathcal{L}$ from (25).

4. For each N, the model with largest log-likelihood $\mathcal{L}$ is selected as the best-fit model.

Supplementary Note 7. SCALING OF COMPUTATIONAL EFFORT

In Supplementary Figure 10, we plot the number of model evaluations used in each search for the best-fit phenomenological model. We define a model evaluation as a single integration of a system of ODEs. (Note that the amount of necessary CPU time per integration is dependent on the size and stiffness of the system.) This includes both integration of model ODEs and the derivatives of model ODEs, used in gradient calculations.[9] Note that in Figure 4, to indicate the total number of evaluations used as N is gradually increased to its final value, we plot the cumulative sum of the number of model evaluations depicted in Supplementary Figure 10. We see that the number of model evaluations scales superlinearly with N if the selected model size is growing with N, as is the case in the yeast glycolysis model below about $N = 20$ (Supplementary Figure 10 and Supplementary Figure 11). When the model size saturates, the number of model evaluations scales roughly linearly with N.

[9] The number of integrations per gradient calculation is proportional to the number of parameters. This means that the computational effort used to fit large models is dominated by gradient calculations.

Supplementary Note 8. COMPARISON TO BAYESIAN NETWORK APPROACHES

A related set of methods for inferring causal structure from time series data comes from the field of Bayesian Networks (BN), and specifically Dynamic Bayesian Networks (dBN). Implementations typically make the following assumptions:

1. Variables are updated at a discrete set of times, rather than continuously.

2. Latent variables are not allowed, or their number is known *a priori*.

3. The state space of dynamical variables is itself discrete (and often of low cardinality, such as binary or ternary).

Many generalizations of (d)BNs have been presented that lift each of these assumptions. Below we include a brief literature review of current implementations of (d)BNs that address each issue. However, our method is distinct from (d)BNs in that it is designed to perform inference simultaneously for *continuous variables*, in *continuous time*, with potentially a *very large number of unknown hidden nodes*, and we are not aware of an approach that is able to lift all three assumptions in order to analyze the type of data handled by *Sir Isaac*.

Continuous time: It is known that exact inference is intractable in continuous time versions of dBNs because calculating a node's distribution at a given time step does not easily factor into conditionally independent subsets, as it does in cases with discrete time. Instead, each node's distribution will in general depend on the entire history of all other variables [18]. Approximate methods have been developed to deal with such Continuous Time Bayesian Networks (CTBNs) [18, 19]. Conversely, converting a set of ODEs, such as those explored by *Sir Isaac*, into the dBN framework generally leads to an exponentially large model [20] that cannot be readily inferred from data.

Continuous states: Most implementations deal with discrete state variables, to avoid the need to infer multidimensional distributions over continuous variables, which can require very large data sets. It is also relatively common to use continuous variables by specifying the state of nodes as finite-parameter continuous distributions, such as Gaussians. However, these differ from

Sir Isaac in that they are typically parameterized with means that are simply linear combinations of parent nodes (e. g., [21, 22]). One approach uses biochemically inspired functions relating means of continuous-valued nodes [23], but does not use continuous time.

Unspecified network size: Though some approaches attempt to discover that hidden nodes are necessary for a better description of a system (e. g., [23–25]), this is not a typical feature of Bayesian network implementations. Approaches that are complete, in the sense that they allow, in principle, infinitely many latent variables, are relatively rare (e. g., [26]), and do not address continuous space-time requirements.

SUPPLEMENTARY REFERENCES

[1] Schmidt, M. *et al.* Automated refinement and inference of analytical models for metabolic networks. *Phys Biol* **8**, 055011 (2011).

[2] Ruoff, P., Christensen, M., Wolf, J. & Heinrich, R. Temperature dependency and temperature compensation in a model of yeast glycolytic oscillations. *Biophys Chem* **106**, 179–192 (2003).

[3] Nemenman, I. Fluctuation-dissipation theorem and models of learning. *Neural Comput* **17**, 2006–2033 (2005).

[4] Savageau, M. A. & Voit, E. O. Recasting Nonlinear Differential Equations as S-Systems: A Canonical Nonlinear Form. *Math Biosci* **87**, 83–115 (1987).

[5] Landau, L. & Lifshitz, E. *Mechanics* (Butterworth-Heinemann, 1976), 3rd edn.

[6] Hlavacek, W. S. *et al.* Rules for modeling signal-transduction systems. *Sci. STKE* **2006**, re6 (2006).

[7] Bionetgen. http://bionetgen.org.

[8] Myers, C. R., Gutenkunst, R. N. & Sethna, J. P. Python unleashed on systems biology. *Computing in Science and Engineering* **9**, 34 (2007).

[9] Gutenkunst, R. N. *et al.* Sloppycell. http://sloppycell.sourceforge.net.

[10] Daniels, B. C. & Nemenman, I. Efficient inference of parsimonious phenomenological models of cellular dynamics using S-systems and alternating regression. *PLoS One* **10**, e0119821 (2015).

[11] Vyshemirsky, V. & Girolami, M. Bayesian ranking of biochemical system models. *Bioinform* **24**, 833–839 (2008).

[12] Toni, T., Welch, D., Strelkowa, N., Ipsen, A. & Stumpf, M. Approximate Bayesian computation scheme for parameter inference and model selection in dynamical systems. *J R Soc Interf* **6**, 187–202 (2009).

[13] Lillacci, G. & Khammash, M. Parameter estimation and model selection in computational biology. *PLoS Comput Biol* **6**, e1000696 (2010).

[14] Balasubramanian, V. Statistical inference, occam's razor, and statistical mechanics on the space of probability distributions. *Neural Comput* **9**, 349–368 (1997).

[15] Bialek, W., Nemenman, I. & Tishby, N. Predictability, complexity, and learning. *Neural Comput* **13**, 2409 (2001).

[16] Schwarz, G. Estimating the dimension of a model. *Annals Stat* **6**, 461–464 (1978).

[17] Wolpert, D. & Macready, W. No free lunch theorems for optimization. *IEEE Transactions on Evolutionary Computation* **1**, 67–82 (1997).

[18] Nodelman, U., Shelton, C. & Koller, D. Continuous time Bayesian networks. *Proceedings of the Eighteenth Conference on Uncertainty in Artificial Intelligence (UAI)* 378–387 (2002). URL `http://dl.acm.org/citation.cfm?id=2073921`.

[19] Nodelman, U. D. *Continuous time Bayesian networks*. Ph.D. thesis, Stanford University (2007).

[20] Chatterjee, S. & Russell, S. Why are DBNs sparse? In *International Conference on Artificial Intelligence and Statistics (AISTATS)*, 81–88 (Sardinia, Italy, 2010). URL `http://machinelearning.wustl.edu/mlpapers/paper\_files/AISTATS2010\_ChatterjeeR10.pdf`.

[21] Friedman, N., Linial, M., Nachman, I. & Pe'er, D. Using Bayesian Networks to Analyze Expression Data. *J Comput Biol* **7**, 601–620 (2000).

[22] Markowetz, F. & Spang, R. Inferring cellular networks–a review. *BMC Bioinf* **8 Suppl 6**, S5 (2007).

[23] Nachman, I., Regev, a. & Friedman, N. Inferring quantitative models of regulatory networks from expression data. *Bioinformatics* **20 Suppl 1**, i248–56 (2004).

[24] Elidan, G., Lotner, N., Friedman, N. & Koller, D. Discovering hidden variables: A structure-based approach. In *Advances in Neural Information Processing Systems (NIPS 2000)* (2000).

[25] Bagrow, J. *et al.* Shadow networks: Discovering hidden nodes with models of information flow. *http://arxiv.org/abs/1312.6122* (2013).

[26] Doshi-Velez, F., Wingate, D., Roy, N., Tenenbaum, J. & Roy, N. Infinite dynamic bayesian networks. In *International Conference on Machine Learning (ICML)* (2011).
